# Non-coding RNAs and gastrointestinal cancers prognosis: an umbrella review of systematic reviews and meta-analyses of observational studies

**DOI:** 10.3389/fonc.2023.1193665

**Published:** 2023-07-20

**Authors:** Bowen Zha, Yuxi Luo, Muladili Kamili, Xiaqin Zha

**Affiliations:** ^1^ The Sixth Clinical Medical College, Capital Medical University, Beijing, China; ^2^ The First Clinical Medical College, Capital Medical University, Beijing, China; ^3^ Department of Blood Purification, University Affiliated Second Hospital, Nanchang, China

**Keywords:** ncRNA, gastrointestinal cancers, prognosis, umbrella review, miRNA

## Abstract

**Aim:**

Provide an overview and a systematic evaluation of the evidence quality on the association between non-coding RNAs (ncRNAs) and prognosis value for gastrointestinal cancers (GICs).

**Methods:**

We searched the literature from three electronic databases: Pubmed, Embase, and Web of science, then carefully screened and extracted the primary information and results from the included articles. We use A measurable systematic review and meta-analysis evaluation tool (AMSTAR2) to evaluate the quality of methodology and then use the Grading of Recommendations Assessment 2, Development and Evaluation guideline (GRADE) make sure the reliability of the meta-analysis.

**Results:**

Overall, 182 meta-analyses from 58 studies were included in this study. Most of these studies are of low or very low quality. Using the scoring tool, we found that only two meta-analyses were rated as high reliability, and 17 meta-analyses were rated as medium reliability.

**Conclusions:**

Although ncRNA has good prognostic value in some studies, only a tiny amount of evidence is highly credible at present. More research is needed in the future.

**PROSPERO registration number:**

CRD42022382296.

## Introduction

With the development of sequencing technology, more and more non-coding RNA has been found (1). NcRNA play an important role in maintaining cell homeostasis and performing multiple functions (2). A study based on colorectal cancer found that the deletion of junctional adhesion molecule A induced by MIR21 promoted the activation and metastasis of oncogenes (3). In addition, ncRNA can affect the body function by affecting other genes. For example, miR-137 can down-regulate glyoxalase 1, while another glyoxalase 1 can affect the immune response (4, 5). Recent study suggests that ncRNA can also affect the occurrence and development of gastric cancer by affecting epigenetics (6).

And now there have been study to develop ncRNA drugs for the targeted treatment of neurodegenerative diseases (7). Some other drugs are also being actively developed (8). In terms of gastrointestinal cancer, a study in 2021 recommended three miRNA molecules as potential therapeutic targets (9). Another study suggests that part of ncRNA can be used as chemosensitivity regulator to assist patients in chemotherapy (10). Moreover, some stable ncRNA are also used to predict the prognosis of the disease (11, 12).

Gastrointestinal tumors (GICs), such as colorectal cancer (CRC), esophageal cancer (EC), stomach cancer (SC), liver cancer (LC), and pancreas cancer (PC), are the leading causes of cancer-related deaths worldwide (13). At present, many studies have revealed the prognostic effect of some ncRNA on GICs (14, 15). And some meta-analysis on the prognostic effect of ncRNA on GICs has been published (16). We aim to make regression evaluations, find high-quality evidence, and provide a basis for future research.

## Methods

This study followed the Preferred Reporting Items for Systematic Reviews and Meta-Analyses (PRISMA) guidelines (17), and the study protocol has been registered in the PROSPERO (CRD42022382296).

### Search strategy

We search literature from Pubmed, Web of Science, and Embase databases. Search time is from the establishment of the database to January 2023. The details of search words are listed in **Supplementary Table 1**. Then we manually searched for references of relevant articles to identify potentially eligible studies. Two researchers independently screened titles and abstracts. If there were any differences, we would discuss them until achieving a consensus.

### Inclusion and exclusion criteria

The inclusion criteria were as follows (1): assessing the role of ncRNA in the prognosis of GIC (2); providing at least one prognostic outcome data (overall survival, disease-free survival, progression-free survival, and recurrence-free survival) (3); containing meta-analysis in the study.

The exclusion criteria were as follows (1): focus on genetics or experiments not in humans (2); full-text not available (3); lack critical information.

If two or more eligible studies evaluate the prognostic value of the same ncRNA for the same disease, we will include the largest number of original studies.

### Data extraction

The first author, year of publication, journal, disease, kind of ncRNA, number of studies, total population, results, and tools for assessing the risk of bias were extracted. At the same time, we extract the number of studies, number of participants, relative risk, P value, I^2^, effect model, and publication bias in each meta-analysis. If the study carries out subgroup analysis according to the types of ncRNA, we will extract the relevant results. Two researchers independently extracted the data and cross-verified it.

### Evaluation of the quality of the study

A Measurement Tool to Assess systematic Reviews 2 (AMSTAR2) is a questionnaire that asks reviewers to answer ‘yes,’ ‘partly yes,’ or ‘no’ (18). Among the total 16 items, seven items are considered the most important when assessing the quality of meta-analyses: registration in advance, reasonable search strategy, reasonable exclusion of literature, adoption of appropriate bias evaluation tools, selection of appropriate statistical methods, consideration of the impact of bias on results, and consideration of potential bias risks of articles. In the study, we viewed two ‘part yes’ as one ‘yes.’ According to the evaluation of 16 items, the final results are evaluated as high, moderate, low, or critically low. Two researchers independently evaluate the quality and cross-verified it. For visual display, we use Python to make forest maps to reveal the prognostic value of ncRNAs to GICs.

### Grading of the evidence of meta-analysis

Grading of Recommendations Assessment, Development, and Evaluation (GRADE) methods propose five factors rating down certainty in the evidence (the risk of bias, inconsistency, indirectness, imprecision, and publication bias) and two factors rating up certainty in the evidence (large effect and dose-response) (19, 20). Based on GRADE, outcomes are evaluated as high, moderate, low, or very low quality. As for prognostic studies, the quality of evidence was high, and we downgraded and upgraded them by five rating down factors and two rating up factors, respectively (21). Only the standardized, systematic evaluation of research reports is suitable for grading the results, so we do not grade the research results rated as extremely low quality by ASMTAR2. Two researchers completed this step independently. Moreover, the results were shown by corresponding scales using Python.

## Results

### Study selection

**Figure 1:**
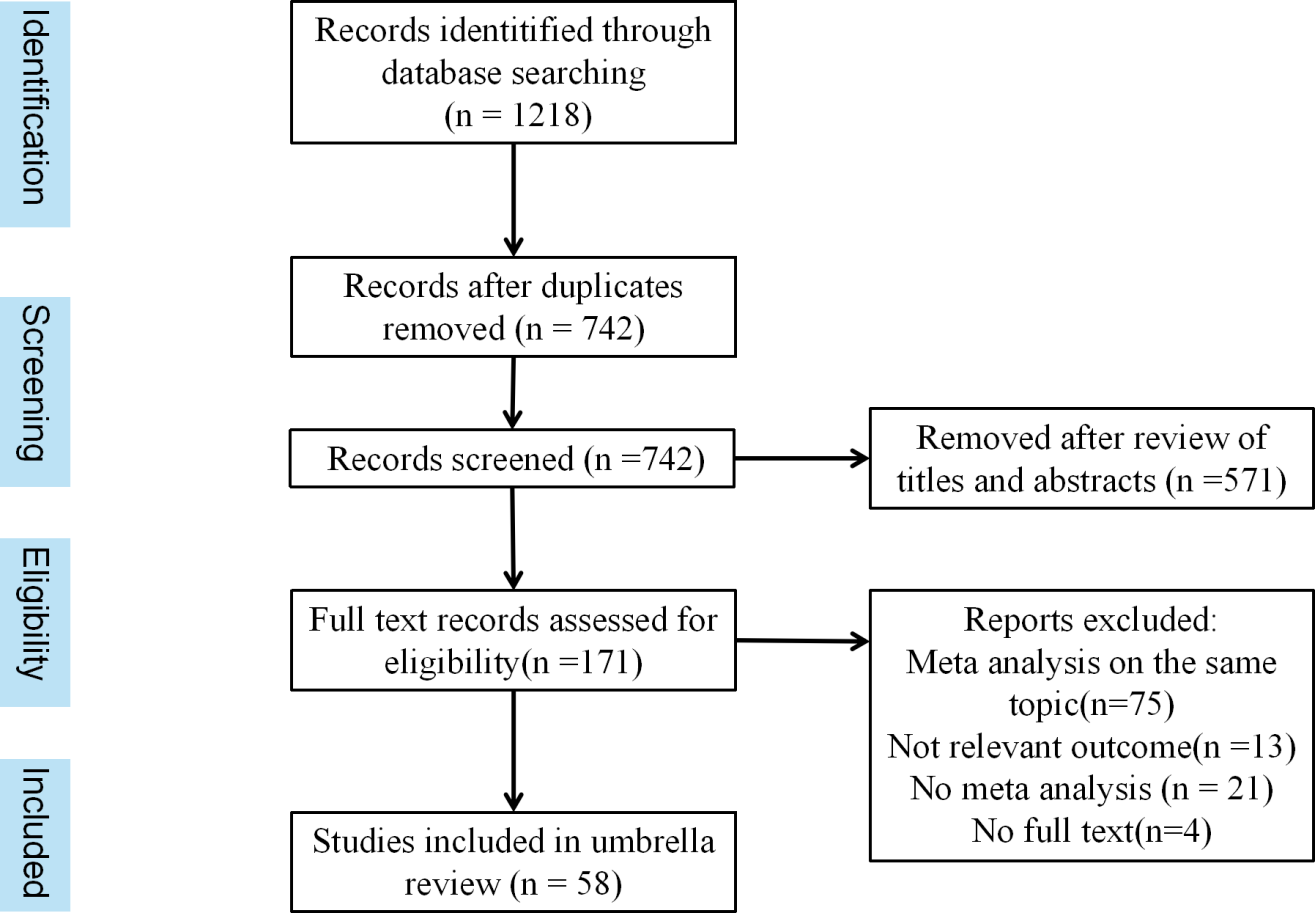
Flowchart of systematic review and meta-analysis selection.

Overall, 1218 articles were retrieved from three databases. After removing 742 duplicates and screening the titles and abstracts, 171 articles were identified. A further 113 articles were excluded during the full-text reading for the following reasons: 13 articles had no relevant outcome, 21 articles did not perform a meta-analysis, 4 studies have no full text, and 75 articles discussed the same topic. Ultimately, 58 studies were included in this umbrella review. As shown in **Figure 1**.

### Colorectal cancer

CRC is a research hotspot. There are 25 studies to analyze the prognostic value of ncRNA for CRC (22–46). One study analyzed circRNAs, while Another study analyzed ciR-7 separately (36, 42). One study included 42 original studies for meta-analysis for lncRNA (25), and the remaining seven studies were analyzed for different lncRNA. In addition, 23 different miRNAs were analyzed in 15 studies. Details can be found in **Table 1**, **Supplementary Table 2** and **Supplementary Figure 1**.

**Table 1:** Characteristics of the prognosis meta-analyses with methodological quality. .

Journal	Biomarker	No of studies in each MA	No of Participants	Tools for assessing the risk of the bias	Disease	Outcomes
Biomed Res Int	circRNA ciRs-7	13	1714	NOS	CRC, SC	OS
BMC Cancer	circRNAs	8	690	NOS	CRC	OS
Cancer Manag Res	lncRNA CRNDE	5	679	NOS	CRC	OS
Open Med	lncRNA HOTAIR	6	629	NOS	CRC	OS, RFS
Aging	lncRNA HULC	14	1312	NOS	CRC, SC, LC, PC	OS
Oncol Lett	lncRNA MALAT1	12	1157	NOS	CRC, LC	OS
Front Oncol	lncRNA MTA1	27	2954	NOS	CRC, EC	OS
Cell Physiol Biochem	lncRNA TUBA4B	23	3109	NOS	CRC	OS
Medicine	lncRNA UCA1	7	775	NOS	CRC	OS
Dis Markers	lncRNA	111	13103	MOOSE	CRC, EC, SC	OS
Biomed Res Int	miR-15a	4	863	NOS	CRC	OS
Cancers	miR-20a	5	1170	Other	CRC	OS
Cancer Manag Res	miR-21; miR-92a; miR-125b; miR-126; miR-181a; miR-429	63	10254	NR	CRC	OS,DFS
Cancer Cell Int	miR-29	4	437	NOS	CRC	OS
BMC Cancer	miR-106	22	2954	NOS	CRC	OS, DFS
Dis Markers	miR-124	29	3061	NOS	CRC,SC, LC, PC	OS
Pathol Oncol Res	miR-133	10	1340	NOS	CRC, SC	OS
J Cancer	miR-141	3	801	NOS	CRC	OS
Int J Biol Markers	miR-143; miR-145	17	5128	NOS	CRC	OS
J Pers Med	miR-150	3	397	QUADAS2	CRC	OS
Cancer Med	miR-181	9	1017	NOS	CRC	OS
Cancer Cell Int	miR-200a; miR-200b; miR-200c	30	9027	NOS	CRC	OS
Onco Targets Ther	miR-203	9	1258	NOS	CRC	OS
Arch Med Res	miR-224	22	3000	Other	CRC, SC, LC	OS
Oncotarget	miR-494	15	1104	MOOSE	CRC, PC	OS
Cancer Med	circRNAs	6	572	NOS	EC	OS
Clin Chim Acta	lncRNA HOTAR	5	510	Other	EC	OS
J Genet	lncRNA AK001796; lncRNA Casc9; lncRNA LINC00460; lncRNA MEG3; lncRNA PCAT-1; lncRNA UCA1; lncRNA MALAT1; lncRNA XIST	51	6510	NOS	EC	OS
Clin Transl Gastroenterol	Let-9g; miR-9; miR-16, miR-21; miR-26a; miR-34a, miR-92a; miR-100; miR-133a; miR-133b; miR-138; miR-143-3p; miR-145; miR-155; miR-200; miR-203; miR-205; miR-223; miR-455-3p; miR-655	44	4310	NOS	EC	OS
Eur Arch Otorhinolaryngol	miR-375	8	934	MOOSE	EC	OS
Cancer Med	circRNAs	35	3135	NOS	SC	OS
Medicine	lncRNA HOTAIR	11	876	NOS	SC	OS
Cancer Med	lncRNA AFAP1-AS1	5	493	NR	SC	OS
Front Oncol	lncRNA FOXP4-AS1	2	408	NOS	SC	OS
Dis Markers	lncRNA PVT1	8	747	NOS	SC	OS
Sci Rep	lncRNA TP73-AS1	5	270	NOS	SC	OS
Onco Targets Ther	lncRNA ANRIL; lncRNA CASCI5; lncRNA CCAT2; lncRNA GAPLING; lncRNA H19; lncRNA HOTTIP; lncRNA LINC00673; lncRNA Malat1; lncRNA MEG3; lncRNA PANDAR; lncRNA Sox2ot; lncRNA SPRY4-ITI; lncRNA UCA1; lncRNA XIST; lncRNA ZEBI-AS1; lncRNA ZFAS1	51	6095	NR	SC	OS
Medicine	miR-10b	4	768	NOS	SC	OS, DFS
Gastroenterol Hepatol Bed Bench	miR-125	10	1203	NOS	SC	OS
Open Med	miR-92a	3	593	NOS	SC	OS
Dis Markers	miR-200c	7	935	QUADAS	SC	OS
Med Sci Monit	miR-21	5	351	NR	SC	OS
Oncotarget	miR-20a; miR-20b; miR-27b; miR-34a; miR-106b; miR-107; miR-137; miR-141; miR-143; miR-146a; miR-150; miR-183; miR-192; miR-196a; miR-196b; miR-206; miR-214; miR-218; miR-335; miR-451; miR-506	69	6148	Other	SC	OS
Cancer Epidemiol Biomarkers Prev	miR-145	4	640	NOS	SC	OS, PFS
Medicine	miR-125-5p	3	455	NOS	SC	OS
Cancer Metastasis Rev	miR-181	2	72	NR	SC	OS
J Transl Med	mIR-130	24	2141	NOS	SC, LC	OS
Can J Gastroenterol Hepatol	circRNAs	10	1090	NOS	LC	OS
Medicine	lncRNA SNHG16	3	257	NOS	LC	OS
J Cell Physiol	lncRNA HOTAIR	2	124	NOS	LC	OS
Oncotarget	lncRNAC PVT1	2	303	NOS	LC	OS
Oncotarget	lncRNA UCA1	14	1441	NOS	LC, PC	OS
J Healthc Eng	lncRNA	29	4670	NOS	LC	OS, RFS, DPS
Medicine	miR-122	11	1124	NOS	LC	OS
Int J Biol Markers	miR-221	7	416	NOS	LC	OS
Mol Aspects Med	miR-141; miR-200	58	8107	NR	LC, PC	OS
Cell Physiol Biochem	miR-203	2	214	NOS	LC	OS
Aging	miR-21; miR-196a; miR-451a; miR-1290; miR-10b; miR-17-5p; miR-23a; miR-29c; miR-126; miR-155; miR-200c; miR-203; miR-218; miR-221; miR-222	57	5445	NOS	PC	OS

DFS, Disease free-survival; OS, Overall survival; RFS, Recurrence-free survival; NOS, The Newcastle-Ottawa Scale; MOOSE, Meta-analysis of Observational Studies in Epidemiology; Other, The author uses other evaluation tools; QUADAS, Quality Assessment of Diagnostic Accuracy Studies; EC, Esophageal carcinoma; SC, stomach cancer; LC, liver carcinoma; CRC, colorectal cancer; PC, pancreatic cancer; NR, No report.

Of the 25 studies, ten were considered critically low methodological quality. The main defects are no protocol or guidance literature and insufficient consideration of the risk of bias included in the study. We further use GRADE to evaluate the meta-analysis of the remaining studies. Among them, lncRNA HOTAIR is considered highly credible, and it has no serious problems in the five degradation factors. It has an upgrade factor of a large magnitude of effect. While circRNA(up), ciRs-7, lncRNA CRNDE, lncRNA UCA1, miR-124, and miR-203 are considered moderate credibility, they have one or two problems in reducing factors. At the same time, circRNA(down), lncRNA MALAT1, and miR-133 are considered to have low credibility. Other studies have extremely low credibility. The detailed evaluation process is in the **Supplementary Materials**.

MiR-203 has no significant relationship with CRC in the medium or high-reliability meta-analysis. The other six analyses have a significant relationship, as shown in **Supplementary Table 3**.

### Esophagus cancer

Seven studies have reported the prognostic value of ncRNA for EC, including four reports of lncRNA, two reports of miRNA, and one report of circRNA (25, 28, 47–51). Two study made subgroup analyses of lncRNA and miRNA, respectively, and analyzed their prognostic value according to the types of ncRNA (47, 49). Details can be found in **Table 1**, **Supplementary Table 2** and **Supplementary Figure 2**.

According to ASMTAR2, one study was considered critically low quality because two important indicators have not been met (28). Other studies further evaluate the credibility of their meta-analysis according GRADE. Among them, circRNAs, lncRNA HOTAR, lncRNA AK001796, lncRNA Casc9, and lncRNA MEG3 are considered as moderate credibility. Although they have problems in continuity or directness, they are rated as medium because of large magnitude of effect. LncRNA MEG3 was negatively correlated with EC prognosis, and the other four were positively correlated with EC prognosis. In addition, lncRNA Linc00460, lncRNA PCAT-1 and lncRNA UCA1 are considered to be of low reliability. The other 25 meta-analyses are of extremely low reliability. The detailed evaluation process is in the **Supplementary Materials**.

### Stomach cancer

As another research hotspot, 23 studies about SC were included in this study (25, 27, 36, 44–46, 52–68). Two studies related to circRNA (36, 53). One study conducted a detailed subgroup analysis according to the types of lncRNAs (55), and other eight studies also analyzed different lncRNAs. The remaining 11 studies are all related to miRNA. Fifteen studies used NOS to evaluate the quality of included literature, and eight studies used other methods or were not reported. Details can be found in **Table 1**, **Supplementary Table 2** and **Supplementary Figure 3**.

Due to the failure to meet the requirements of some critical items, only 12 studies were rated as low quality or above. After carefully using GRADE, we found that circRNAs (up), circRNAs (down), ciRs-7, and lncRNA PVT1 were considered moderately credible. Among them, circRNAs (down) are negatively correlated with the prognosis of SC, while other ncRNAs are positively correlated with the prognosis of SC. Meta-analysis without high-level credibility. MiR-125a, miR-125b, and miR-145 are rated as extremely low credibility, and their evidence is insufficient. Other meta-analyses are low credibility. The detailed evaluation process is in the **Supplementary Materials**.

### Liver cancer

Seven studies each reported the prognostic value of miRNA and lncRNA for LC (27, 37, 44, 45, 60, 69–77), and one reported the circRNA (78). Among them, one study included the most original research, including 29 original studies for meta-analysis (75). Details can be found in **Table 1**, **Supplementary Table 2** and **Supplementary Figure 4**.

Five studies were considered to be of critically low quality. One study did not meet one unimportant item but met the other 15 items, so it was considered high quality. The remaining nine studies were of low quality. In the reliability evaluation of meta-analysis, although there are some problems in precision, lncRNA SNHG16 is rated as high reliability because it meets the upgrade conditions. circRNAs(up) is considered as moderate credibility. The remaining ten meta-analyses are of low or extremely low reliability. The detailed evaluation process is in the **Supplementary Materials**.

### Pancreas cancer

Two studies have reported the prognostic value of lncRNA for PC respectively (27, 71). At the same time, four other studies reported the situation of miRNA (38, 45, 70, 79). At present, we have not found any research on circRNA. Details can be found in **Table 1**, **Supplementary Table 2** and **Supplementary Figure 5**.

Four studies were rated as low quality and above. According to GRADE, only miR-21 was rated as medium quality, and the other 20 meta-analyses were rated as low quality or extremely low quality due to different degradation factors. The detailed evaluation process is in the **Supplementary Materials**.

## Discussion

With the development of gene technology, the treatment of cancer patients has been improved (80, 81). Immunotherapy plays a vital role in cancer treatment (82–84). Recent studies suggest that some ncRNA is related to the immune infiltration of various tumors (85, 86). NcRNA can be used not only as a potential target site, but also as a prognostic indicator.

**Figure 2:**
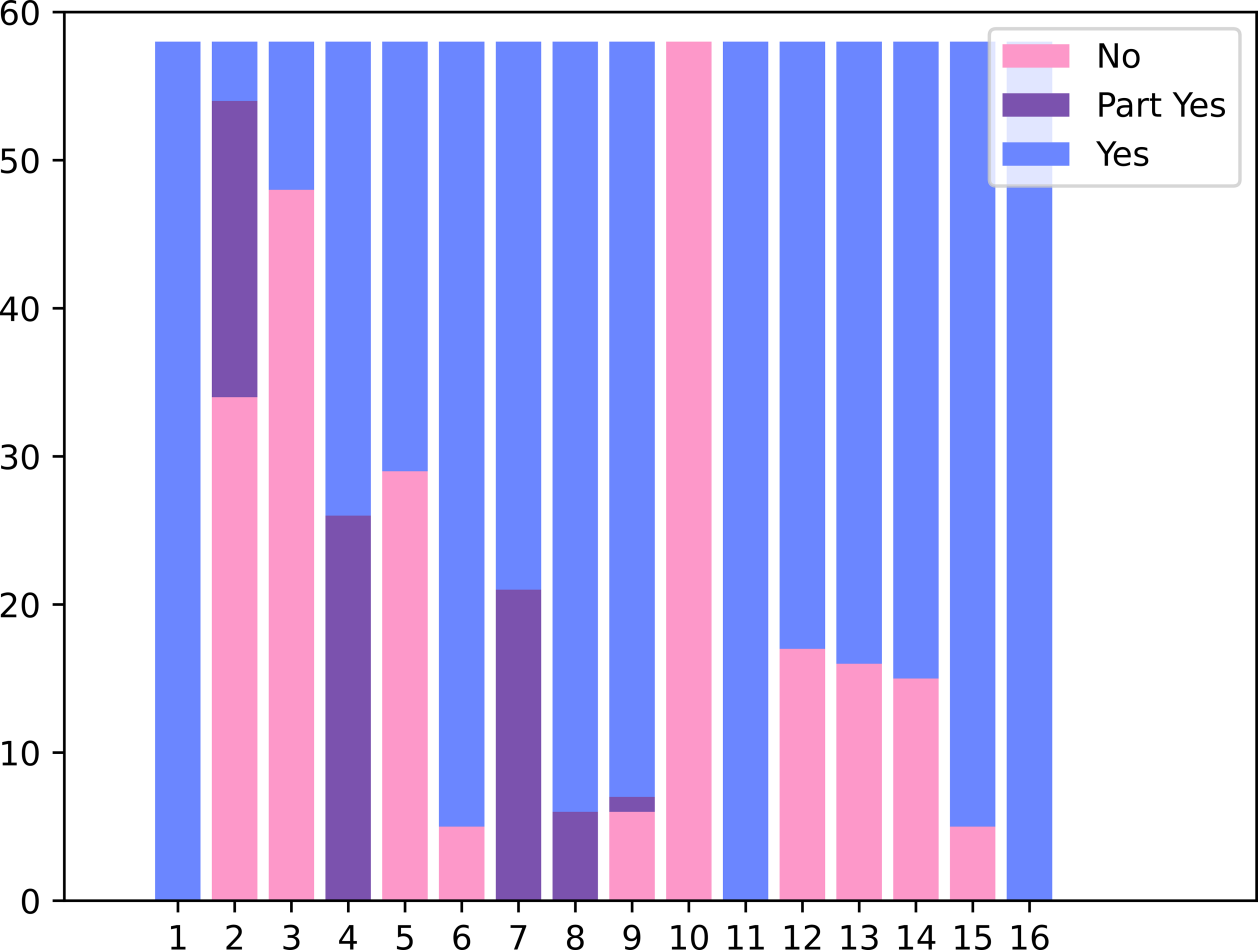
Evaluation of the methodological quality with AMSTAR2.

Among the fifty-eight included studies, one and three were rated as high and moderate quality, 32 were graded as low quality, and 22 were evaluated as critically low quality. For detail of items, no meta-analysis discussed the fund of original studies, while most articles were best done in stem one and item sixteen. The main flaw of included prognostic studies was no protocol or guidance literature. The details can be found in **Figure 2** and **Supplementary Table 3**.

**Figure 3:**
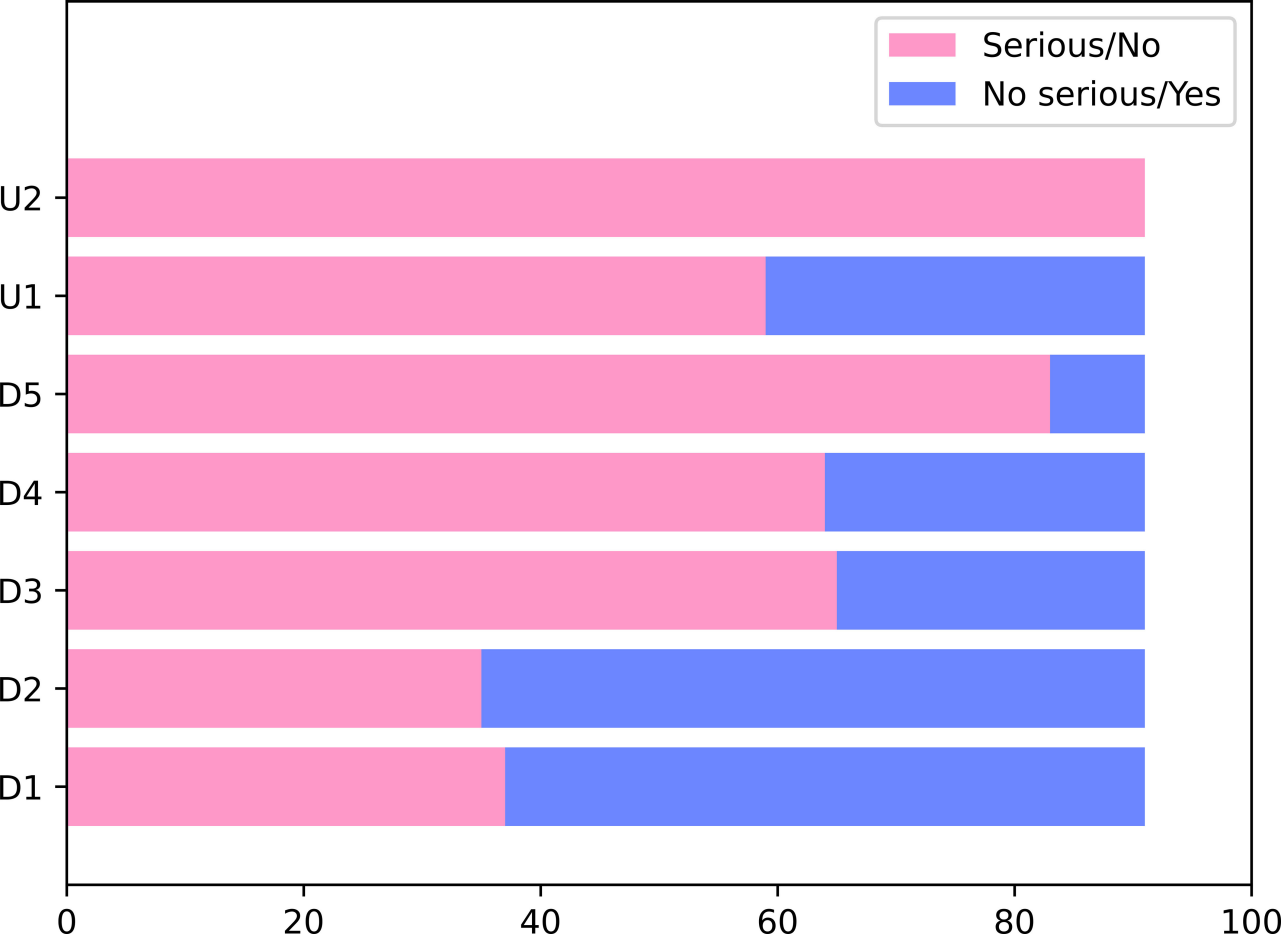
Evaluation of the outcome quality with GRADE.

Apart from critically low-rate studies, we evaluated 91 meta-analyses in the 36 other studies by GRADE. Two meta-analyses were graded as high, 17 were rated as moderate, 21 were supported by low, and 51 meta-analyses presented very low evidence. For down factors, the main flaw of meta-analyses was publication bias, and the best item in the research was inconsistency. As for up factors, part of the meta-analyses met the item of the large magnitude of effect. The prognosis meta-analysis with high or moderate credibility is shown in **Table 2**. The details can be found in **Figure 3** and **Supplementary Table 4**.

**Table 2:** Main findings of the prognosis meta-analysis with high or moderate credibility.

Disease	Biomarker	Relative risk (95% CI)
CRC	ciRs-7	1.95(1.34, 2.84)
CRC	circRNA(up)	2.29(1.50, 3.52)
CRC	lncRNA CRNDE	2.12(1.59, 2.84)
CRC	lncRNA HOTAIR	2.46(1.82, 3.32)
CRC	lncRNA UCA1	2.25(1.77, 2.87)
CRC	miR-124	0.20 (0.08, 0.50)
CRC	miR-203	1.62(0.93, 2.82)
EC	circRNAs	2.25(1.71, 2.95)
EC	lncRNA HOTAR	2.37(1.80, 3.11)
EC	lncRNA AK001796	3.08(1.81, 5.25)
EC	lncRNA Casc9	2.10(1.47, 3.00)
EC	lncRNA MEG3	0.46(0.25, 0.85)
SC	circRNAs(up)	1.83(1.64,2.03)
SC	circRNAs(down)	0.54(0.45, 0.66)
SC	ciRs-7	2.32 (1.48, 3.64)
SC	lncRNA PVT1	1.68(1.43, 1.97)
LC	circRNAs(up)	3.67 (2.07, 6.48)
LC	lncRNA SNHG16	2.10(1.22, 3.60)
PC	miR-21	1.90(1.61, 2.25)

EC, Esophageal carcinoma; SC, stomach cancer; LC, liver carcinoma; CRC, colorectal cancer; PC, pancreatic cancer.

This review is the first attempt to evaluate the published evidence about the prognosis of ncRNAs for GICs. The PRISMA principle is strictly followed in this study in the analysis process. The critical steps of the research, such as literature retrieval, information extraction, article evaluation, and result grading, are all handled by two authors in a double-blind way to reduce subjective differences.

This study has the following limitations. First, the sample sources, detection methods, and critical values of some original studies included in the meta-analysis differ. In this regard, the limited number of studies makes it difficult for us to conduct subgroup analysis, which can only reduce their credibility. Secondly, we only included the research published in the database and did not consider other literature sources. Thirdly, some studies combined ncRNA with other prognostic markers, which we failed to consider in depth in the article.

## Conclusion

The existing evidence shows that part of ncRNA has high prognostic value for GICs. However, on the whole, most of the evidence at present has low credibility. Limited by research quality, heterogeneity, and small research effect. Further research is needed to overcome the limitations of existing evidence.

## Data availability statement

The original contributions presented in the study are included in the article/**Supplementary Material**. Further inquiries can be directed to the corresponding author.

## Author contributions

XZ and BZ designed the study. YL and MK performed the literature search and selected eligible articles. BZ and MK extracted the data. YL and XZ analyzed the data. BZ wrote the first draft of the manuscript and edited the manuscript. All authors contributed to the article and approved the submitted version.
