# Supplementary material for: Non-coding RNAs and gastrointestinal cancers prognosis: an umbrella review of systematic reviews and meta-analyses of observational studies
Source: Front Oncol. 2023 Jul 20;13:1193665. doi: 10.3389/fonc.2023.1193665 (PMC10399243; doi:10.3389/fonc.2023.1193665)
Supplement: Supplementary file 1 [file DataSheet_1.doc]

**Supplementary Material**

Supplementary Table 1: Search term

Supplementary Table 2: Main findings of the progonsis of ncRNA in cancer

Supplementary Table 3: Detailed evaluation of the progonsis with AMSTAR 2

Supplementary Table 4: Detailed evaluation of the methodological quality of the progonsis oucomes with GRADE

Supplementary Table 5: PRISMA checklist

Supplementary Figure 1: Main findings of the progonsis of ncRNA for CRC(Overall survival)

Supplementary Figure 2: Main findings of the progonsis of ncRNA for EC(Overall survival)

Supplementary Figure 3: Main findings of the progonsis of ncRNA for SC(Overall survival)

Supplementary Figure 4: Main findings of the progonsis of ncRNA for LC(Overall survival)

Supplementary Figure 5: Main findings of the progonsis of ncRNA for PC(Overall survival)

Supplementary Table 1: Search term

| **#** | **Searches** | **Results** |
| --- | --- | --- |
| **PubMed** | | |
| 1 | "Systematic Review" [Publication Type] | 221497 |
| 2 | "Meta-Analysis" [Publication Type] | 176304 |
| 3 | #1 OR #2 | 302216 |
| 4 | "Stomach cancer" [MeSH Terms] OR "Stomach cancer" [Title/Abstract] | 111215 |
| 5 | "Esophagus cancer" [MeSH Terms] OR "Esophagus cancer" [Title/Abstract] | 58305 |
| 6 | "Colorectal cancer" [MeSH Terms]OR "Colorectal cancer" [Title/Abstract] | 263811 |
| 7 | "Pancreas cancer" [MeSH Terms]OR "Pancreas cancer" [Title/Abstract] | 89911 |
| 8 | "Liver cancer"[MeSH Terms]OR "liver cancer" [Title/Abstract] | 201202 |
| 9 | #4 OR #5 OR #6 OR #7 OR #8 | 680370 |
| 10 | "Prognosis"[Title/Abstract] OR "Prognostic" [Title/Abstract] | 756639 |
| 11 | "NcRNA" [MeSH Terms]OR "NcRNA" [Title/Abstract] | 247571 |
| 12 | "MiRNA" [MeSH Terms]OR "MiRNA" [Title/Abstract] | 139901 |
| 13 | "LncRNA" [MeSH Terms]OR "LncRNA" [Title/Abstract] | 41388 |
| 14 | "CircRNA" [MeSH Terms]OR "CircRNA" [Title/Abstract] | 9819 |
| 15 | "SnRNA" [MeSH Terms]OR "SnRNA" [Title/Abstract] | 5891 |
| 16 | #11 OR #12 OR #13 OR #14 OR #15 | 276870 |
| 17 | #3 OR #9 | 12050 |
| 18 | #10 AND #17 | 2146 |
| 19 | #16 OR #18 | 148 |
| **Embase** | | |
| 1 | 'meta analysis'/exp | 265782 |
| 2 | 'systematic review'/exp | 385314 |
| 3 | #1 OR #2 | 505127 |
| 4 | 'gastric cancer'/exp OR 'gastric cancer':ti,ab,kw | 149629 |
| 5 | 'esophagus cancer'/exp OR 'esophagus cancer':ti,ab,kw | 86004 |
| 6 | 'colorectal cancer'/exp OR 'colorectal cancerr':ti,ab,kw | 402724 |
| 7 | 'pancreas cancer'/exp OR 'pancreas cancer':ti,ab,kw | 133966 |
| 8 | 'liver cancer'/exp OR 'liver cancer':ti,ab,kw | 316519 |
| 9 | #4 OR #5 OR #6 OR #7 OR #8 | 950699 |
| 10 | 'prognosis':ti,ab,kw OR 'prognostic':ti,ab,kw | 1137989 |
| 11 | 'ncrna'/exp OR ncrna:ti,ab,kw | 445990 |
| 12 | 'mirna'/exp OR mirna:ti,ab,kw | 222128 |
| 13 | 'lncrna'/exp OR lncna:ti,ab,kw | 48280 |
| 14 | 'circrna'/exp OR circna:ti,ab,kw | 6932 |
| 15 | 'snrna'/exp OR 'snrna':ti,ab,kw | 6348 |
| 16 | #11 OR #12 OR#13 OR #14 OR #15 | 452227 |
| 17 | #3 AND #9 AND #10 AND #16 | 341 |

| **WOS** | | |
| --- | --- | --- |
| 1 | (TS= (meta analysis)) OR (TS=(system review)) | 1934826 |
| 2 | (TS= (prognosis)) OR (TS=(prognostic) | 1401655 |
| 3 | ((((TS=ncRNA)) OR TS=(miRNA)) OR TS=(lncRNA)) OR TS=(circRNA)) OR TS=(snRNA) | 143435 |
| 4 | ((((TS=(gastric cancer)) OR TS=(esophagus cancer)) OR TS=(colorectal cancerr)) OR TS=(pancreas cancer)) OR TS=(liver cancer) | 574518 |
| 5 | #1 AND #2 AND #3 AND #4 | 729 |

Supplementary Table 2: Main findings of the progonsis of ncRNA in cancer

| Disease | Biomarker | Metaanalysis metric | No of studies in each MA | No of Participants | Relative risk (95% ci) | P value | I^2^ | Effect model | Publication bias | Study |
| --- | --- | --- | --- | --- | --- | --- | --- | --- | --- | --- |
| CRC | ciRs-7 | OS | 3 | 500 | 1.95(1.34, 2.84) | <0.001 | 0 | Fixed | NR | Tian 2020 |
| CRC | circRNA(up) | OS | 6 | 543 | 2.29(1.50, 3.52) | <0.001 | 0 | Fixed | A | Yuan 2020 |
| CRC | circRNA(down) | OS | 2 | 147 | 0.37(0.22, 0.64) | <0.001 | 0 | Fixed | A | Yuan 2020 |
| CRC | lncRNA CRNDE | OS | 5 | 679 | 2.12(1.59, 2.84) | < 0.001 | 37 | Fixed | NR | He 2019 |
| CRC | lncRNA HOTAIR | OS | 6 | 629 | 2.46(1.82, 3.32) | < 0.01 | 21 | NR | A | Chen 2020 |
| CRC | lncRNA HOTAIR | RFS | 2 | 272 | 1.97(1.27, 3.05) | < 0.01 | 50 | NR | A | Chen 2020 |
| CRC | lncRNA HULC | OS | 3 | 154 | 1.37(0.38, 4.88) | 0.63 | 75 | Random | NR | Li 2022 |
| CRC | lncRNA MALAT1 | OS | 2 | 214 | 3.04 (1.77, 4.31) | < 0.001 | 0 | Fixed | NR | Wang 2019 |
| CRC | lncRNA MTA1 | OS | 2 | 175 | 2.01 (1.39, 2.92) | 0.001 | 0 | Fixed | NR | Li 2020 |
| CRC | lncRNA TUBA4B | OS | 3 | 474 | 1.57 (1.10,2.23) | 0.042 | 42 | Fixed | NR | Zhang 2018 |
| CRC | lncRNA TUBA4B | DFS | 5 | 716 | 1.45 (1.16, 1.82) | 0.003 | 15 | Fixed | NR | Zhang 2018 |
| CRC | lncRNA UCA1 | OS | 7 | 775 | 2.25(1.77, 2.87) | <0.00001 | 0 | Fixed | A | Liu 2019 |
| CRC | lncRNAs | OS | 42 | 5008 | 2.10 (1.87, 2.36) | <0.0001 | 52 | Random | A | Kang 2018 |
| CRC | miR-15a | OS | 4 | 863 | 1.27 (0.69, 2.36) | NR | 71 | Random | NR | Yang 2019 |
| CRC | miR-20a | OS | 5 | 1170 | 2.02 (0.90, 3.14) | NR | 69 | Random | NR | Moody 2019 |
| CRC | miR-21 | OS | 13 | 2861 | 1.31(1.12, 1.53) | < 0.01 | 65 | Random | NR | Gao 2018 |
| CRC | miR-21 | DFS | 7 | 554 | 1.64(1.11, 2.41) | < 0.01 | 72 | Random | NR | Gao 2018 |
| CRC | miR-29 | OS | 4 | 437 | 0.69(0.39, 1.25) | 0.22 | 86 | Random | NR | Peng 2019 |
| CRC | miR-92a | OS | 2 | 240 | 2.11(0.59, 7.61) | 0.25 | 82 | Random | NR | Gao 2018 |
| CRC | miR-106a | OS | 9 | 1088 | 1.45(1.16, 1.80) | 0.001 | 49 | Fixed | NR | Peng 2020 |
| CRC | miR-106b | OS | 2 | 276 | 1.57 (0.35, 7.08) | 0.555 | 81 | Random | NR | Peng 2020 |
| CRC | miR-106a | DFS | 6 | 693 | 1.44(0.88, 2.35) | 0.151 | 80 | Random | NR | Peng 2020 |
| CRC | miR-106b | DFS | 5 | 897 | 2.19(1.61, 3.00) | <0.001 | 0 | Fixed | NR | Peng 2020 |
| CRC | miR-124 | OS | 2 | 145 | 0.20 (0.08, 0.50) | 0.763 | 0 | Fixed | NR | Zhou 2019 |
| CRC | miR-125b | OS | 5 | 857 | 1.43(0.83, 2.47) | 0.19 | 75 | Random | NR | Gao 2018 |
| CRC | miR-126 | OS | 5 | 948 | 1.55(1.24, 1.93) | < 0.01 | 1 | Random | NR | Gao 2018 |
| CRC | miR-133 | OS | 2 | 272 | 0.80(0.25, 2.60) | 0.708 | 84 | Random | NR | Zhu 2020 |
| CRC | miR-141 | OS | 3 | 801 | 1.58(0.78-3.19) | NR | 64 | Random | NR | Yu 2019 |
| CRC | miR-143 | OS | 7 | 2472 | 0.98(0.59, 1.63) | 0.943 | 78 | Random | A | Li 2019 |
| CRC | miR-145 | OS | 6 | 1604 | 1.92(1.45, 2.54) | 0.000 | 26 | Fixed | A | Li 2019 |
| CRC | miR-150 | OS | 3 | 397 | 1.05(0.57, 1.94) | 0.86 | 83 | Random | NR | Sur 2020 |
| CRC | miR-181a | OS | 3 | 665 | 1.52 (1.26, 1.83) | <0.01 | 0 | Random | NR | Gao 2018 |
| CRC | miR-181 | OS | 9 | 1017 | 1.75(1.26, 2.43) | <0.05 | 67 | Random | A | Peng 2019 |
| CRC | miR-200a | OS | 10 | 3352 | 1.13 (1.01, 1.26) | 0.027 | 70 | Random | NR | Peng 2020 |
| CRC | miR-200b | OS | 9 | 3163 | 1.14 (0.97, 1.33) | 0.113 | 72 | Random | NR | Peng 2020 |
| CRC | miR-200c | OS | 11 | 2512 | 1.00 (0.85, 1.17) | 0.973 | 74 | Random | NR | Peng 2020 |
| CRC | miR-203 | OS | 9 | 1258 | 1.62(0.93, 2.82) | 0.09 | 83 | Random | A | Ye 2017 |
| CRC | miR-224 | OS | 9 | 848 | 2.27(1.30, 3.97) | <0.01 | 69 | Random | A | Zhang 2017 |
| CRC | miR-429 | OS | 5 | 467 | 1.00( 0.39, 2.58) | 1.00 | 89 | Random | NR | Gao 2018 |
| CRC | miR-494 | OS | 2 | 351 | 2.59(1.62, 4.14) | NR | 0 | Fixed | NR | Xiang 2018 |
| EC | circRNAs | OS | 6 | 572 | 2.25(1.71, 2.95) | 0 | 0 | Fixed | A | Guo 2020 |
| EC | lncRNA HOTAR | OS | 5 | 510 | 2.37( 1.80, 3.11) | <0.00001 | 0 | Random | NR | Song 2016 |
| EC | lncRNA AK001796 | OS | 2 | 225 | 3.08(1.81, 5.25) | NR | 0 | Fixed | NR | Qian 2020 |
| EC | lncRNA Casc9 | OS | 3 | 288 | 2.10(1.47, 3.00) | NR | 0 | Fixed | NR | Qian 2020 |
| EC | lncRNA Llnc00460 | OS | 2 | 142 | 3.49(1.88, 6.48) | NR | 0 | Fixed | NR | Qian 2020 |
| EC | lncRNA MEG3 | OS | 2 | 239 | 0.46(0.25, 0.85) | NR | 0 | Fixed | NR | Qian 2020 |
| EC | lncRNA PCAT-1 | OS | 2 | 451 | 1.77(1.24, 2.54) | NR | 0 | Fixed | NR | Qian 2020 |
| EC | lncRNA UCA1 | OS | 3 | 256 | 1.96(1.33, 2.87) | NR | 12 | Fixed | NR | Qian 2020 |
| EC | lncRNA MALAT1 | OS | 4 | 339 | 1.74(1.33, 2.28) | NR | 80 | Random | NR | Qian 2020 |
| EC | lncRNA XIST | OS | 2 | 267 | 1.78(1.31, 2.43) | NR | 53 | Random | NR | Qian 2020 |
| EC | lncRNA MTA1 | OS | 7 | 786 | 1.55(1.14, 2.11) | 0.006 | 82 | Random | NR | Li 2020 |
| EC | lncRNAs | OS | 23 | 3123 | 1.92 (1.70, 2.16) | <0.00001 | 21 | Random | A | Kang 2018 |
| EC | let-9g | OS | 2 | 197 | 1.27(0.66, 2.45) | 0.47 | 59 | Random | NR | Gao 2019 |
| EC | miR-9 | OS | 2 | 342 | 1.07(0.45, 2.57) | 0.88 | 73 | Random | NR | Gao 2019 |
| EC | miR-21 | OS | 10 | 1071 | 1.63(1.26, 2.11) | <0.01 | 24 | Random | NR | Gao 2019 |
| EC | miR-26a | OS | 2 | 116 | 1.09(0.19, 6.39) | 0.92 | 48 | Random | NR | Gao 2019 |
| EC | miR-34a | OS | 2 | 210 | 1.87(0.88, 3.99) | 0.11 | 45 | Random | NR | Gao 2019 |
| EC | miR-92a | OS | 2 | 170 | 1.47(0.64, 3.34) | 0.36 | 54 | Random | NR | Gao 2019 |
| EC | miR-100 | OS | 4 | 410 | 2.12(0.86, 5.21) | 0.1 | 73 | Random | NR | Gao 2019 |
| EC | miR-133a | OS | 2 | 210 | 2.48(1.50, 4.12) | <0.01 | 0 | Random | NR | Gao 2019 |
| EC | miR-133b | OS | 2 | 265 | 2.15(1.27, 3.62) | <0.01 | 0 | Random | NR | Gao 2019 |
| EC | miR-138 | OS | 2 | 333 | 2.27(1.68, 3.08) | <0.01 | 0 | Random | NR | Gao 2019 |
| EC | miR-143-3p | OS | 2 | 199 | 1.12(0.13, 9.33) | 0.92 | 95 | Random | NR | Gao 2019 |
| EC | miR-145 | OS | 2 | 143 | 0.85(0.27, 2.66) | 0.79 | 73 | Random | NR | Gao 2019 |
| EC | miR-155 | OS | 2 | 283 | 1.17(0.64, 2.14) | 0.61 | 48 | Random | NR | Gao 2019 |
| EC | miR-200a | OS | 2 | 187 | 0.71(0.19, 2.60) | 0.60 | 79 | Random | NR | Gao 2019 |
| EC | miR-203 | OS | 2 | 70 | 2.83(1.35, 5.96) | <0.01 | 0 | Random | NR | Gao 2019 |
| EC | miR-205 | OS | 2 | 57 | 0.75(0.09, 6.45) | 0.79 | 72 | Random | NR | Gao 2019 |
| EC | miR-223 | OS | 2 | 294 | 1.13(0.25, 5.03) | 0.87 | 65 | Random | NR | Gao 2019 |
| EC | miR-455-3p | OS | 2 | 326 | 0.67(0.10, 4.48) | 0.68 | 94 | Random | NR | Gao 2019 |
| EC | miR-655 | OS | 2 | 63 | 2.66(1.16, 6.12) | 0.02 | 0 | Random | NR | Gao 2019 |
| EC | miR-16 | OS | 2 | 62 | 1.23(0.14, 10.86) | 0.86 | 90 | Random | NR | Gao 2019 |
| EC | miR-25 | OS | 2 | 257 | 1.75(0.56, 5.54) | 0.34 | 67 | Random | NR | Gao 2019 |
| EC | miR-375 | OS | 8 | 934 | 1.58 (1.29, 1.94) | <0.0001 | 6 | Fixed | NR | Wang 2019 |
| SC | circRNAs(up) | OS | 22 | 1802 | 1.83(1.64,2.03) | <0.00001 | 0 | Fixed | A | Chen 2019 |
| SC | circRNAs(downr) | OS | 13 | 1333 | 0.54(0.45, 0.66) | <0.00001 | 0 | Fixed | A | Chen 2019 |
| SC | circRNAs(up) | DFS | 5 | 377 | 2.63(2.08, 3.33) | <0.00001 | 0 | Fixed | NR | Chen 2019 |
| SC | circRNAs(downr) | DFS | 5 | 814 | 0.39(0.27, 0.59) | <0.00001 | 0 | Fixed | NR | Chen 2019 |
| SC | ciRs-7 | OS | 2 | 256 | 2.32 (1.48, 3.64) | <0.001 | 0 | Fixed | NR | Tian 2020 |
| SC | lncRNA | OS | 40 | 4972 | 1.96 (1.77, 2.16) | <0.00001 | 21 | Random | A | Kang 2018 |
| SC | lncRNA MTA1 | OS | 2 | 547 | 2.08(1.80, 2.41) | <0.001 | 0 | Fixed | NR | Li 2020 |
| SC | lncRNA HOTAIR | OS | 11 | 876 | 1.81 (1.52, 2.15) | <0.001 | 16 | Fixed | NR | Zhang 2018 |
| SC | lncRNA AFAP1-AS1 | OS | 5 | 493 | 2.49(2.02, 3.08) | <0.00001 | 11 | Fixed | NR | Duan 2022 |
| SC | lncRNA PVT1 | OS | 5 | 598 | 1.68(1.43, 1.97) | <0.001 | 0 | Fixed | NR | Hao 2021 |
| SC | lncRNA PVT1 | DFS | 5 | 598 | 1.74(1.44, 2.08) | <0.001 | 0 | Fixed | NR | Hao 2021 |
| SC | lncRNA ANRIL | OS | 2 | 220 | 1.68(1.16, 2.43) | <0.001 | 0 | Random | NR | Gao 2018 |
| SC | lncRNA CASCI5 | OS | 2 | 148 | 1.99(1.21, 2.28) | <0.001 | 0 | Random | NR | Gao 2018 |
| SC | lncRNA CCAT2 | OS | 2 | 193 | 2.17(1.53, 3.09) | <0.001 | 0 | Random | NR | Gao 2018 |
| SC | lncRNA GAPLING | OS | 2 | 123 | 1.49(1.18, 1.89) | <0.001 | 0 | Random | NR | Gao 2018 |
| SC | lncRNA H19 | OS | 4 | 643 | 1.51(1.05, 2.17) | 0.03 | 64 | Random | NR | Gao 2018 |
| SC | lncRNA HOTTIP | OS | 3 | 540 | 1.57(1.20, 2.05) | <0.001 | 0 | Random | NR | Gao 2018 |
| SC | lncRNA LINC00673 | OS | 2 | 152 | 2.47(1.45, 4.20) | <0.001 | 0 | Random | NR | Gao 2018 |
| SC | lncRNA Malat1 | OS | 4 | 496 | 1.70(1.33, 2.18) | <0.001 | 30 | Random | NR | Gao 2018 |
| SC | lncRNA MEG3 | OS | 2 | 206 | 1.96(1.17, 3.28) | <0.001 | 0 | Random | NR | Gao 2018 |
| SC | lncRNA PANDAR | OS | 2 | 246 | 3.11(2.72, 3.55) | <0.001 | 0 | Random | NR | Gao 2018 |
| SC | lncRNA Sox2ot | OS | 2 | 287 | 2.30(1.52, 3.46) | <0.001 | 0 | Random | NR | Gao 2018 |
| SC | lncRNA SPRY4-ITI | OS | 2 | 236 | 1.42(0.48, 4.22) | 0.53 | 71 | Random | NR | Gao 2018 |
| SC | lncRNA UCA1 | OS | 4 | 399 | 1.73(1.12, 2.68) | 0.01 | 46 | Random | NR | Gao 2018 |
| SC | lncRNA XIST | OS | 2 | 204 | 1.89(1.38, 2.59) | <0.001 | 23 | Random | NR | Gao 2018 |
| SC | lncRNA ZEBI-AS1 | OS | 2 | 831 | 2.07(1.67, 2.56) | <0.001 | 0 | Random | NR | Gao 2018 |
| SC | lncRNA ZFAS1 | OS | 2 | 158 | 2.51(1.34, 4.69) | <0.001 | 0 | Random | NR | Gao 2018 |
| SC | lncRNA FOXP4-AS1 | OS | 2 | 408 | 0.89(0.68, 1.16) | 0.381 | 0 | Fixed | NR | Zhang 2022 |
| SC | lncRNA TP73-AS1 | OS | 4 | 270 | 2.10(1.52, 2.91) | NR | 5 | Fixed | NR | Zhong 2020 |
| SC | lncRNA HULC | OS | 2 | 142 | 3.35(1.53, 7.37) | 0.003 | 48 | Fixed | NR | Li 2022 |
| SC | miR-10b | OS | 4 | 768 | 1.57(1.24, 1.99) | <0.001 | 47 | Random | NR | Mei 2020 |
| SC | miR-10b | DFS | 2 | 255 | 1.50 (0.80, 2.82) | 0.212 | 40 | Random | NR | Mei 2020 |
| SC | miR-125a | OS | 8 | 829 | 1.70(1.38, 2.08) | <0.001 | NR | NR | NR | Amiri 2021 |
| SC | miR-125b | OS | 2 | 374 | 1.16( 0.71, 1.89) | 0.53 | NR | NR | NR | Amiri 2021 |
| SC | miR-92a | OS | 3 | 593 | 1.37(0.92, 3.24) | NA | 81 | Random | NR | Guo 2021 |
| SC | miR-200c | OS | 7 | 935 | 2.19 (1.51, 3.17) | <0.01 | 47 | Random | A | Huang 2019 |
| SC | miR-200c | PFS | 3 | 479 | 1.64(0.93, 2.89) | 0.086 | 61 | NR | NR | Huang 2019 |
| SC | miR-200c | DFS | 3 | 358 | 1.73(1.33, 2.33) | <0.01 | 0 | NR | NR | Huang 2019 |
| SC | miR-21 | OS | 5 | 351 | 2.00 (1.39, 2.88) | <0.001 | 38 | Fixed | A | Wang 2014 |
| SC | miR-20a | OS | 3 | 199 | 1.25(0.84-1.87) | 0.27 | 71 | Random | NR | Zhang 2017 |
| SC | miR-20b | OS | 3 | 178 | 2.38(1.16-4.87) | 0.02 | 0 | Random | NR | Zhang 2017 |
| SC | miR-27b | OS | 3 | 319 | 1.18(0.75-1.85) | 0.47 | 36 | Random | NR | Zhang 2017 |
| SC | miR-34a | OS | 5 | 457 | 1.25(0.59-2.65) | 0.56 | 68 | Random | NR | Zhang 2017 |
| SC | miR-106b | OS | 2 | 157 | 1.84(1.15-2.94) | 0.01 | 0 | Random | NR | Zhang 2017 |
| SC | miR-107 | OS | 3 | 248 | 1.52(0.42-5.57) | 0.52 | 89 | Random | NR | Zhang 2017 |
| SC | miR-137 | OS | 2 | 168 | 3.21(1.68-3.37) | <0.01 | 6 | Random | NR | Zhang 2017 |
| SC | miR-141 | OS | 2 | 125 | 2.47(1.34-4.56) | <0.01 | 0 | Random | NR | Zhang 2017 |
| SC | miR-143 | OS | 2 | 81 | 0.68(0.12, 3.81) | 0.66 | 49 | Random | NR | Zhang 2017 |
| SC | miR-146a | OS | 3 | 213 | 2.60(1.63, 4.13) | <0.01 | 14 | Random | NR | Zhang 2017 |
| SC | miR-150 | OS | 3 | 223 | 1.63(0.77, 3.45) | 0.2 | 48 | Random | NR | Zhang 2017 |
| SC | miR-183 | OS | 3 | 478 | 1.46(0.55, 3.83) | 0.45 | 90 | Random | NR | Zhang 2017 |
| SC | miR-192 | OS | 3 | 140 | 1.71(0.60, 4.85) | 0.31 | 87 | Random | NR | Zhang 2017 |
| SC | miR-196a | OS | 4 | 286 | 2.66(1.94, 3.63) | <0.01 | 0 | Random | NR | Zhang 2017 |
| SC | miR-196b | OS | 4 | 625 | 1.67(1.38, 2.02) | <0.01 | 0 | Random | NR | Zhang 2017 |
| SC | miR-206 | OS | 3 | 468 | 2.85(1.73, 4.70) | <0.01 | 0 | Random | NR | Zhang 2017 |
| SC | miR-214 | OS | 4 | 403 | 1.84(1.27, 2.67) | <0.01 | 23 | Random | NR | Zhang 2017 |
| SC | miR-218 | OS | 3 | 220 | 2.61(1.74, 3.92) | <0.01 | 0 | Random | NR | Zhang 2017 |
| SC | miR-335 | OS | 2 | 124 | 0.85(0.03, 27.50) | 0.93 | 95 | Random | NR | Zhang 2017 |
| SC | miR-451 | OS | 3 | 332 | 1.73(1.19, 2.52) | <0.01 | 15 | Random | NR | Zhang 2017 |
| SC | miR-506 | OS | 3 | 288 | 2.07(1.33, 3.23) | <0.01 | 0 | Random | NR | Zhang 2017 |
| SC | miR-224 | OS | 2 | 201 | 1.82(1.14, 2.95) | <0.01 | 0 | Random | NR | Zhang 2017 |
| SC | miR-145 | OS | 4 | 640 | 1.40(0.79, 2.48) | 0.24 | 77 | Random | NR | Xu 2019 |
| SC | miR-133a | OS | 2 | 537 | 0.54(0.39, 0.76) | <0.001 | 0 | Fixed | NR | Zhu 2020 |
| SC | miR-124 | OS | 2 | 209 | 0.46 (0.31, 0.68) | 0.549 | 0 | Fixed | NR | Zhou 2019 |
| SC | miR-125-5p | OS | 3 | 455 | 0.34 (0.16, 0.73) | 0.005 | 52 | Random | NR | Ye 2019 |
| SC | miR-181 | OS | 2 | 72 | 0.67(0.20, 2.19) | NR | NR | NR | NR | Pop-Bica 2018 |
| SC | miR-130a | OS | 3 | 658 | 1.81 (1.34, 2.45) | < 0.001 | 0 | Fixed | NR | Peng 2019 |
| LC | circRNAs(up) | OS | 4 | 279 | 3.67 (2.07, 6.48) | <0.001 | 47 | Fixed | NR | Hao 2019 |
| LC | circRNAs(down) | OS | 6 | 811 | 0.38 (0.30, 0.48) | <0.001 | 0 | Fixed | NR | Hao 2019 |
| LC | lncRNA SNHG16 | OS | 3 | 257 | 2.10(1.22, 3.60) | 0.007 | 17 | Fixed | A | Liu 2021 |
| LC | lncRNA SNHG1 | DFS | 2 | 169 | 3.38(1.10, 10.40) | 0.03 | 84 | Random | A | Liu 2021 |
| LC | lncRNA HoTAL | OS | 2 | 124 | 3.43 (1.93, 6.12) | NR | 0 | Fixed | NR | Abdeahad 2018 |
| LC | lncRNA MALAT1 | OS | 2 | 92 | 1.46 (0.76, 2.17) | <0.001 | 0 | Fixed | NR | Wang 2019 |
| LC | lncRNAC PVT1 | OS | 2 | 303 | 1.11(0.66, 1.57) | NR | 0 | Fixed | NR | Zhu 2018 |
| LC | lncRNA UCA1 | OS | 2 | 338 | 1.89(0.96, 2.82) | <0.001 | 0 | Fixed | NR | Liu 2017 |
| LC | lncRNA HULC | OS | 3 | 436 | 0.86(0.44-1.66) | 0.65 | 80 | Random | NR | Li 2022 |
| LC | lncRNA | OS | 29 | 4670 | 2.32(2.08, 2.59) | <0.01 | 0 | Fixed | NR | Wang 2021 |
| LC | lncRNA | RFS | 4 | 867 | 2.19(1.72, 2.78) | <0.01 | 0 | Fixed | NR | Wang 2021 |
| LC | lncRNA | DFS | 7 | 1145 | 1.88(1.57, 2.25) | <0.01 | 38 | Fixed | NR | Wang 2021 |
| LC | miR-122 | OS | 4 | 328 | 1.48(1.22, 1.80) | <0.001 | 34 | Fixed | NR | Zhang 2019 |
| LC | miR-122 | PFS | 7 | 796 | 1.54(1.28, 1.85) | <0.001 | 83 | Random | NR | Zhang 2019 |
| LC | miR-221 | OS | 7 | 416 | 1.91(1.53, 2.38) | <0.01 | 0 | Fixed | A | Liu 2021 |
| LC | miR-124 | OS | 3 | 307 | 0.56 (0.40, 0.78) | 0.049 | 67 | Random | NR | Zhou 2019 |
| LC | miR-200 | OS | 3 | 557 | 2.10(0.60, 7.31) | 0.246 | NR | Random | NR | Huang 2019 |
| LC | miR-203 | OS | 2 | 214 | 0.59(0.43, 0.82) | 0.002 | 49 | Fixed | NR | Shao 2017 |
| LC | miR-130b | OS | 4 | 357 | 2.43 (1.28,4.63) | 0.004 | 72 | Random | NR | Peng 2019 |
| LC | miR-224 | OS | 2 | 318 | 0.86(0.30, 2.50) | 0.78 | 91 | Random | NR | Zhang 2017 |
| PC | lncRNA UCA1 | OS | 2 | 208 | 1.58(1.01, 2.15) | <0.001 | 0 | Fixed | NR | Liu 2017 |
| PC | lncRNA HULC | OS | 2 | 364 | 4.58(1.57, 13.37) | 0.005 | 72 | Random | NR | Li 2022 |
| PC | miR-124 | OS | 2 | 118 | 0.55 (0.35, 0.86) | 0.171 | 47 | Fixed | NR | Zhou 2019 |
| PC | miR-200 | OS | 5 | 395 | 0.52 (0.25, 1.09) | 0.082 | NR | Random | NR | Huang 2019 |
| PC | miR-141 | OS | 2 | 134 | 0.28 (0.10, 0.73) | 0.009 | NR | Random | NR | Huang 2019 |
| PC | miR-494 | OS | 3 | 236 | 0.47 ( 0.33, 0.68) | NR | 0 | Fixed | NR | Xiang 2018 |
| PC | miR-196a | OS | 2 | 66 | 1.61(0.50, 5.23) | 0.43 | 80 | Random | NR | Zhao2020 |
| PC | miR-451a | OS | 3 | 137 | 2.23(1.23, 4.04) | <0.01 | 2 | Random | NR | Zhao2020 |
| PC | miR-1290 | OS | 2 | 223 | 1.43(1.04, 1.95) | 0.03 | 0 | Random | NR | Zhao2020 |
| PC | miR-10b | OS | 4 | 375 | 1.73(1.09, 2.76) | 0.02 | 62 | Random | NR | Zhao2020 |
| PC | miR-17-5p | OS | 3 | 164 | 1.91(1.30, 2.80) | <0.01 | 0 | Random | NR | Zhao2020 |
| PC | miR-21 | OS | 19 | 1947 | 1.90(1.61, 2.25) | <0.01 | 44 | Random | A | Zhao2020 |
| PC | miR-23a | OS | 4 | 251 | 2.18(1.52, 3.13) | <0.01 | 0 | Random | NR | Zhao2020 |
| PC | miR-29c | OS | 4 | 463 | 1.39(1.08, 1.79) | 0.01 | 52 | Random | NR | Zhao2020 |
| PC | miR-126 | OS | 3 | 455 | 1.55(1.23, 1.95) | <0.01 | 0 | Random | NR | Zhao2020 |
| PC | miR-155 | OS | 3 | 211 | 2.22(1.27, 3.88) | <0.01 | 0 | Random | NR | Zhao2020 |
| PC | miR-200c | OS | 3 | 258 | 1.40(0.51, 3.79) | 0.51 | 87 | Random | NR | Zhao2020 |
| PC | miR-203 | OS | 4 | 619 | 1.65(1.14, 2.40) | <0.01 | 84 | Random | NR | Zhao2020 |
| PC | miR-218 | OS | 3 | 248 | 2.62(1.41, 4.88) | <0.01 | 58 | Random | NR | Zhao2020 |
| PC | miR-221 | OS | 4 | 187 | 1.72(1.08, 2.74) | 0.02 | 5 | Random | NR | Zhao2020 |
| PC | miR-222 | OS | 3 | 322 | 1.72(1.02, 2.91) | 0.04 | 37 | Random | NR | Zhao2020 |

NR: No report; A:No obvious bias; B: Have obvious bias; CRC, coloectal cancer; EC,esophagus cancer; SC, stomach cancer; LC, liver cancer; PC, pancreas cancer.

Supplementary Table 3: Detailed evaluation of the methodological quality of the progonsis with AMSTAR 2

| Study | 1 | 2 | 3 | 4 | 5 | 6 | 7 | 8 | 9 | 10 | 11 | 12 | 13 | 14 | 15 | 16 | Final rating |
| --- | --- | --- | --- | --- | --- | --- | --- | --- | --- | --- | --- | --- | --- | --- | --- | --- | --- |
| CRC | | | | | | | | | | | | | | | | | |
| Tian 2020 | Y | pY | N | Y | Y | Y | pY | Y | Y | N | Y | Y | Y | Y | Y | Y | Low |
| Yuan 2020 | Y | N | Y | pY | Y | Y | Y | pY | Y | N | Y | Y | Y | Y | Y | Y | Low |
| He 2019 | Y | N | N | Y | Y | Y | pY | Y | Y | N | Y | Y | Y | Y | Y | Y | Low |
| Chen 2019 | Y | pY | N | Y | N | Y | pY | Y | Y | N | Y | Y | Y | Y | Y | Y | Low |
| Li 2022 | Y | N | N | pY | Y | Y | pY | Y | Y | N | Y | Y | Y | Y | Y | Y | Critically low |
| Wang 2019 | Y | N | N | Y | N | Y | Y | Y | Y | N | Y | Y | Y | Y | Y | Y | Low |
| Li 2020 | Y | N | N | pY | Y | Y | Y | pY | Y | N | Y | N | N | Y | Y | Y | Critically low |
| Zhang 2018 | Y | N | N | pY | N | Y | pY | Y | Y | N | Y | Y | N | Y | Y | Y | Critically low |
| Liu2019 | Y | N | N | Y | N | Y | pY | Y | Y | N | Y | Y | Y | Y | Y | Y | Low |
| Kang 2018 | Y | N | N | pY | Y | Y | Y | Y | Y | N | Y | Y | Y | Y | Y | Y | Low |
| Yang 2019 | Y | pY | N | Y | N | Y | pY | Y | Y | N | Y | N | Y | N | Y | Y | Low |
| Moody 2019 | Y | N | Y | Y | Y | Y | pY | Y | Y | N | Y | N | Y | N | Y | Y | Low |
| Gao 2018 | Y | N | N | pY | Y | Y | Y | Y | N | N | Y | Y | N | N | Y | Y | Critically low |
| Peng 2019 | Y | N | N | Y | N | Y | pY | Y | Y | N | Y | Y | Y | Y | Y | Y | Low |
| Peng 2020 | Y | N | N | Y | N | Y | pY | Y | Y | N | Y | Y | N | Y | Y | Y | Critically low |
| Zhou 2019 | Y | N | N | pY | N | Y | Y | Y | Y | N | Y | Y | Y | Y | Y | Y | Low |
| Zhu 2020 | Y | N | N | Y | Y | Y | Y | Y | Y | N | Y | Y | Y | Y | Y | Y | Low |
| Yu 2019 | Y | N | N | Y | N | Y | pY | Y | Y | N | Y | Y | Y | Y | Y | Y | Low |
| Li 2019 | Y | pY | N | pY | N | Y | Y | pY | Y | N | Y | Y | N | N | Y | Y | Critically low |
| Sur 2020 | Y | pY | Y | pY | Y | Y | pY | Y | pY | N | Y | N | N | N | N | Y | Critically low |
| Peng 2019 | Y | pY | N | Y | Y | Y | pY | Y | Y | N | Y | N | N | N | Y | Y | Critically low |
| Peng 2020 | Y | pY | N | Y | N | Y | pY | Y | Y | N | Y | N | N | Y | Y | Y | Critically low |
| Ye 2017 | Y | Y | N | Y | N | Y | Y | Y | Y | N | Y | Y | Y | Y | Y | Y | Moderate |
| Zhang 2017 | Y | N | N | pY | N | Y | pY | Y | Y | N | Y | Y | N | Y | Y | Y | Critically low |
| Xiang 2018 | Y | pY | N | Y | N | Y | Y | Y | Y | N | Y | Y | N | Y | Y | Y | Low |
| EC | | | | | | | | | | | | | | | | | |
| Guo 2020 | Y | pY | Y | pY | Y | Y | Y | Y | Y | N | Y | Y | Y | Y | Y | Y | Low |
| Song 2016 | Y | N | N | Y | N | Y | pY | Y | Y | N | Y | Y | Y | Y | Y | Y | Low |
| Qian 2020 | Y | Y | N | pY | N | Y | Y | Y | Y | N | Y | Y | Y | Y | Y | Y | Moderate |
| Gao 2019 | Y | N | N | pY | Y | Y | Y | pY | Y | N | Y | Y | Y | N | Y | Y | Low |
| Wang 2019 | Y | pY | N | Y | N | Y | Y | Y | Y | N | Y | Y | Y | Y | Y | Y | Low |
| SC |  |  |  |  |  |  |  |  |  |  |  |  |  |  |  |  |  |
| Chen 2020 | Y | N | N | pY | N | Y | Y | Y | Y | N | Y | Y | Y | Y | Y | Y | Low |
| Zhang 2018 | Y | N | N | Y | N | Y | Y | pY | Y | N | Y | Y | Y | Y | Y | Y | Low |
| Duan 2022 | Y | pY | Y | pY | N | N | Y | Y | N | N | Y | N | Y | N | N | Y | Critically low |
| Zhang 2022 | Y | Y | N | Y | N | Y | Y | Y | Y | N | Y | N | Y | Y | Y | Y | Low |
| Hao 2021 | Y | N | N | pY | Y | Y | Y | Y | Y | N | Y | Y | Y | Y | Y | Y | Low |
| Zhong 2020 | Y | N | N | pY | Y | Y | Y | Y | Y | N | Y | Y | N | Y | Y | Y | Critically low |
| Gao 2018 | Y | N | N | pY | Y | Y | Y | Y | N | N | Y | Y | Y | Y | Y | Y | Critically low |
| Mei 2020 | Y | pY | N | Y | Y | Y | Y | Y | Y | N | Y | N | Y | N | N | Y | Critically low |
| Amiri 2021 | Y | pY | Y | Y | Y | N | Y | Y | Y | N | Y | Y | Y | Y | Y | Y | Moderate |
| Guo 2021 | Y | N | Y | Y | N | Y | Y | Y | Y | N | Y | N | Y | Y | N | Y | Critically low |
| Huang 2019 | Y | pY | N | pY | Y | Y | pY | Y | N | N | Y | Y | Y | Y | Y | Y | Critically low |
| Wang 2014 | Y | N | Y | pY | Y | Y | Y | pY | N | N | Y | N | N | N | Y | Y | Critically low |
| Zhang 2017 | Y | N | N | pY | Y | Y | Y | Y | Y | N | Y | Y | N | Y | Y | Y | Critically low |
| Xu 2019 | Y | N | N | Y | Y | Y | pY | Y | Y | N | Y | Y | Y | Y | Y | Y | Low |
| Ye 2019 | Y | pY | N | Y | N | N | Y | Y | Y | N | Y | N | Y | Y | Y | Y | Low |
| Pop-Bica 2018 | Y | pY | N | pY | N | Y | Y | Y | Y | N | Y | N | N | N | Y | Y | Critically low |
| Peng 2019 | Y | pY | N | Y | N | N | Y | Y | Y | N | Y | Y | Y | Y | Y | Y | Low |
| LC |  |  |  |  |  |  |  |  |  |  |  |  |  |  |  |  |  |
| Hao 2019 | Y | pY | N | Y | Y | N | Y | Y | Y | N | Y | Y | Y | Y | Y | Y | Low |
| Liu 2021 | Y | N | N | pY | N | Y | Y | Y | Y | N | Y | N | Y | N | Y | Y | Low |
| Abdeahad 2018 | Y | N | N | Y | Y | Y | Y | Y | Y | N | Y | N | N | N | Y | Y | Critically low |
| Zhu 2018 | Y | N | N | pY | Y | Y | Y | Y | Y | N | Y | Y | Y | Y | Y | Y | Low |
| Liu 2017 | Y | N | N | Y | N | Y | Y | Y | Y | N | Y | N | Y | Y | Y | Y | Low |
| Wang 2021 | Y | N | Y | Y | N | Y | pY | Y | Y | N | Y | Y | Y | Y | N | Y | Critically low |
| Zhang 2019 | Y | N | N | Y | Y | Y | pY | Y | Y | N | Y | Y | Y | Y | Y | Y | Low |
| Liu 2021 | Y | Y | Y | Y | Y | Y | Y | Y | Y | N | Y | Y | Y | Y | Y | Y | High |
| Huang 2019 | Y | pY | N | pY | N | Y | Y | Y | N | N | Y | Y | N | Y | Y | Y | Critically low |
| Shao 2017 | Y | pY | N | Y | Y | Y | pY | Y | Y | N | Y | N | Y | N | Y | Y | Low |
| PC |  |  |  |  |  |  |  |  |  |  |  |  |  |  |  |  |  |
| Zhao 2020 | Y | N | N | pY | Y | Y | Y | Y | Y | N | Y | Y | Y | N | Y | Y | Low |

Y: Yes; pY: Part Yes; N: no; CRC, coloectal cancer; EC,esophagus cancer; SC, stomach cancer; LC, liver cancer; PC, pancreas cancer.

Supplementary Table 4: Detailed evaluation of thequality of the progonsis oucomes with GRADE

| Disease | Biomaker | Study | Outcomes | Downgrade | | | | | Upgrade | | GRADE |
| --- | --- | --- | --- | --- | --- | --- | --- | --- | --- | --- | --- |
|  |  |  |  | Risk of Bias | lnconsistency | Indirectness | Imprecision | Publication bias | Large magnitude of effect | Dose-response |  |
| CRC | circRNA ciR-7 | Tian 2020 | OS | Serious | No Serious | No Serious | No Serious | Serious | Yes | No | Moderate |
| CRC | circRNAs(up) | Yuan 2020 | OS | No Serious | No Serious | Serious | No Serious | Serious | Yes | No | Moderate |
| CRC | circRNAs(down) | Yuan 2020 | OS | No Serious | No Serious | Serious | Serious | Serious | Yes | No | Low |
| CRC | lncRNA CRNDE | He 2019 | OS | Serious | No Serious | No Serious | No Serious | Serious | Yes | No | Moderate |
| CRC | lncRNA HOTAIR | Chen 2019 | OS | No Serious | No Serious | Serious | No Serious | No Serious | Yes | No | High |
| CRC | lncRNA MALAT1 | Wang 2019 | OS | No Serious | No Serious | Serious | Serious | Serious | Yes | No | Low |
| CRC | lncRNA UCA1 | Liu 2019 | OS | No Serious | No Serious | Serious | No Serious | Serious | Yes | No | Moderate |
| CRC | lncRNAs | Kang 2018 | OS | Serious | Serious | Serious | No Serious | Serious | Yes | No | Very low |
| CRC | miR-15a | Yang 2019 | OS | Serious | Serious | No Serious | No Serious | Serious | No | No | Very low |
| CRC | miR-20a | Moody 2019 | OS | Serious | Serious | Serious | No Serious | Serious | Yes | No | Very low |
| CRC | miR-29 | Peng 2019 | OS | Serious | Serious | Serious | Serious | Serious | No | No | Very low |
| CRC | miR-124 | Zhou 2019 | OS | No Serious | Serious | No Serious | No Serious | Serious | Yes | No | Moderate |
| CRC | miR-133 | Zhu 2020 | OS | No Serious | Serious | No Serious | Serious | Serious | No | No | Low |
| CRC | miR-203 | Ye 2017 | OS | No Serious | Serious | No Serious | No Serious | No Serious | No | No | Moderate |
| CRC | miR-494 | Xiang 2018 | OS | Serious | No Serious | Serious | Serious | Serious | Yes | No | Very low |
| EC | circRNAs | Guo 2020 | OS | Serious | No Serious | Serious | No Serious | No Serious | Yes | No | Moderate |
| EC | lncRNA HOTAR | Song 2016 | OS | No Serious | No Serious | Serious | No Serious | Serious | Yes | No | Moderate |
| EC | lncRNA AK001796 | Qian 2020 | OS | No Serious | No Serious | No Serious | Serious | Serious | Yes | No | Moderate |
| EC | lncRNA Casc9 | Qian 2020 | OS | No Serious | No Serious | No Serious | Serious | Serious | Yes | No | Moderate |
| EC | lncRNA Llnc00460 | Qian 2020 | OS | Serious | No Serious | No Serious | Serious | Serious | Yes | No | Low |
| EC | lncRNA MEG3 | Qian 2020 | OS | No Serious | No Serious | No Serious | Serious | Serious | Yes | No | Moderate |
| EC | lncRNA PCAT-1 | Qian 2020 | OS | No Serious | No Serious | No Serious | Serious | Serious | No | No | Low |
| EC | lncRNA UCA1 | Qian 2020 | OS | No Serious | No Serious | No Serious | Serious | Serious | No | No | Low |
| EC | lncRNA MALAT1 | Qian 2020 | OS | No Serious | Serious | No Serious | Serious | Serious | No | No | Very low |
| EC | lncRNA XIST | Qian 2020 | OS | No Serious | Serious | No Serious | Serious | Serious | No | No | Very low |
| EC | let-9g | Gao 2019 | OS | No Serious | Serious | No Serious | Serious | Serious | No | No | Very low |
| EC | miRNA-9 | Gao 2019 | OS | Serious | Serious | Serious | Serious | Serious | No | No | Very low |
| EC | miRNA-21 | Gao 2019 | OS | Serious | No Serious | Serious | Serious | Serious | No | No | Very low |
| EC | miRNA-26a | Gao 2019 | OS | Serious | No Serious | Serious | Serious | Serious | No | No | Very low |
| EC | miRNA-34a | Gao 2019 | OS | Serious | No Serious | Serious | Serious | Serious | No | No | Very low |
| EC | miRNA-92a | Gao 2019 | OS | Serious | Serious | Serious | Serious | Serious | No | No | Very low |
| EC | miRNA-100 | Gao 2019 | OS | Serious | Serious | Serious | Serious | Serious | Yes | No | Very low |
| EC | miRNA-133a | Gao 2019 | OS | Serious | No Serious | Serious | Serious | Serious | Yes | No | Very low |
| EC | miRNA-133b | Gao 2019 | OS | Serious | No Serious | Serious | Serious | Serious | Yes | No | Very low |
| EC | miRNA-138 | Gao 2019 | OS | Serious | No Serious | Serious | Serious | Serious | Yes | No | Very low |
| EC | miRNA-143-3p | Gao 2019 | OS | Serious | Serious | Serious | Serious | Serious | No | No | Very low |
| EC | miRNA-145 | Gao 2019 | OS | Serious | Serious | Serious | Serious | Serious | No | No | Very low |
| EC | miRNA-155 | Gao 2019 | OS | Serious | No Serious | Serious | Serious | Serious | No | No | Very low |
| EC | miRNA-200a | Gao 2019 | OS | Serious | Serious | Serious | Serious | Serious | No | No | Very low |
| EC | miRNA-203 | Gao 2019 | OS | Serious | No Serious | Serious | Serious | Serious | Yes | No | Very low |
| EC | miRNA-205 | Gao 2019 | OS | Serious | Serious | Serious | Serious | Serious | No | No | Very low |
| EC | miRNA-223 | Gao 2019 | OS | Serious | Serious | Serious | Serious | Serious | No | No | Very low |
| EC | miRNA-455-3p | Gao 2019 | OS | Serious | Serious | Serious | Serious | Serious | No | No | Very low |
| EC | miRNA-655 | Gao 2019 | OS | Serious | No Serious | Serious | Serious | Serious | Yes | No | Very low |
| EC | miRNA-16 | Gao 2019 | OS | Serious | Serious | Serious | Serious | Serious | No | No | Very low |
| EC | miRNA-25 | Gao 2019 | OS | Serious | Serious | Serious | Serious | Serious | No | No | Very low |
| EC | miR-375 | Wang 2019 | OS | No Serious | No Serious | Serious | Serious | Serious | No | No | Very low |
| EC | lncRNA | Kang 2018 | OS | Serious | No Serious | Serious | No Serious | Serious | No | No | Very low |
| SC | circRNAs(up) | Chen 2019 | OS | No Serious | No Serious | Serious | No Serious | No Serious | No | No | Moderate |
| SC | circRNAs(down) | Chen 2019 | OS | No Serious | No Serious | Serious | No Serious | No Serious | No | No | Moderate |
| SC | circRNA ciRs-7 | Tian 2020 | OS | No Serious | No Serious | Serious | No Serious | Serious | Yes | No | Moderate |
| SC | lncRNAs | Kang 2018 | OS | Serious | No Serious | Serious | No Serious | Serious | No | No | Low |
| SC | lncRNA HOTAIR | Zhang 2018 | OS | Serious | No Serious | Serious | No Serious | Serious | No | No | Low |
| SC | lncRNA PVT1 | Hao 2021 | OS | No Serious | No Serious | No Serious | No Serious | Serious | No | No | Moderate |
| SC | miR-125a | AmiRi 2021 | OS | No Serious | Serious | Serious | No Serious | Serious | No | No | Very low |
| SC | miR-125b | AmiRi 2021 | OS | No Serious | Serious | No Serious | Serious | Serious | No | No | Very low |
| SC | miR-145 | Xu 2019 | OS | No Serious | Serious | Serious | No Serious | Serious | No | No | Very low |
| SC | miR-133a | Zhu 2020 | OS | Serious | No Serious | No Serious | No Serious | Serious | No | No | Low |
| SC | miR-124 | Zhou 2019 | OS | No Serious | Serious | Serious | No Serious | Serious | Yes | No | Low |
| SC | miR-125-5p | Ye 2019 | OS | No Serious | Serious | No Serious | Serious | Serious | Yes | No | Low |
| SC | miR-130a | Peng 2019 | OS | No Serious | No Serious | Serious | No Serious | Serious | No | No | Low |
| LC | circRNAs(up) | Hao 2019 | OS | No Serious | No Serious | Serious | Serious | Serious | Yes | No | Moderate |
| LC | circRNAs(down) | Hao 2019 | OS | No Serious | No Serious | Serious | No Serious | Serious | No | No | Low |
| LC | lncRNA SNHG16 | Liu 2021 | OS | No Serious | No Serious | No Serious | Serious | No Serious | Yes | No | High |
| LC | lncRNA MALAT1 | Wang 2019 | OS | No Serious | No Serious | No Serious | Serious | Serious | No | No | Low |
| LC | lncRNAC PVT1 | Zhu 2018 | OS | No Serious | No Serious | No Serious | Serious | Serious | No | No | Low |
| LC | lncRNA UCA1 | Liu 2017 | OS | No Serious | No Serious | No Serious | Serious | Serious | No | No | Low |
| LC | miR-122 | Zhang 2019 | OS | No Serious | No Serious | Serious | Serious | Serious | No | No | Very low |
| LC | miR-124 | Zhou 2019 | OS | No Serious | Serious | Serious | Serious | Serious | No | No | Very low |
| LC | miR-221 | Liu 2021 | OS | No Serious | No Serious | Serious | Serious | No Serious | No | No | Low |
| LC | miR-203 | Shao 2017 | OS | Serious | No Serious | Serious | Serious | Serious | No | No | Very low |
| LC | miR-130b | Peng 2019 | OS | Serious | Serious | Serious | Serious | Serious | No | No | Very low |
| LC | miR-224 | Zhang 2017 | OS | Serious | Serious | No Serious | Serious | Serious | No | No | Very low |
| PC | lncRNA UCA1 | Liu 2017 | OS | No Serious | No Serious | No Serious | Serious | Serious | No | No | Low |
| PC | miR-124 | Zhou 2019 | OS | No Serious | No Serious | No Serious | Serious | Serious | No | No | Low |
| PC | miR-494 | Xiang 2018 | OS | Serious | No Serious | Serious | Serious | Serious | Yes | No | Very low |
| PC | miR-196a | Zhao2020 | OS | No Serious | Serious | Serious | Serious | Serious | No | No | Very low |
| PC | miR-451a | Zhao2020 | OS | No Serious | No Serious | Serious | Serious | Serious | No | No | Very low |
| PC | miR-1290 | Zhao2020 | OS | No Serious | No Serious | Serious | Serious | Serious | No | No | Very low |
| PC | miR-10b | Zhao2020 | OS | No Serious | Serious | Serious | Serious | Serious | No | No | Very low |
| PC | miR-17-5p | Zhao2020 | OS | No Serious | No Serious | Serious | Serious | Serious | No | No | Very low |
| PC | miR-21 | Zhao2020 | OS | No Serious | No Serious | Serious | No Serious | No Serious | No | No | Moderate |
| PC | miR-23a | Zhao2020 | OS | No Serious | No Serious | Serious | Serious | Serious | Yes | No | Low |
| PC | miR-29c | Zhao2020 | OS | No Serious | Serious | Serious | Serious | Serious | No | No | Very low |
| PC | miR-126 | Zhao2020 | OS | No Serious | No Serious | Serious | Serious | Serious | No | No | Very low |
| PC | miR-155 | Zhao2020 | OS | No Serious | No Serious | Serious | Serious | Serious | Yes | No | Low |
| PC | miR-200c | Zhao2020 | OS | No Serious | Serious | Serious | Serious | Serious | No | No | Very low |
| PC | miR-203 | Zhao2020 | OS | No Serious | Serious | Serious | No Serious | Serious | No | No | Very low |
| PC | miR-218 | Zhao2020 | OS | No Serious | Serious | Serious | Serious | Serious | Yes | No | Very low |
| PC | miR-221 | Zhao2020 | OS | No Serious | No Serious | Serious | Serious | Serious | No | No | Very low |
| PC | miR-222 | Zhao2020 | OS | No Serious | No Serious | Serious | Serious | Serious | No | No | Very low |
| OS: overall survival; CRC, coloectal cancer; EC,esophagus cancer; SC, stomach cancer; LC, liver cancer; PC, pancreas cancer.   1. Risk of Bias: We assigned ‘serious’ when studies with a Newcastle-Ottawa scale score < 7 comprised a large proportion. In order to ensure the reliability of the results, if the study does not report Newcastle-Ottawa scale score, we will set the results as serious. 2. Imprecision: We assigned ‘imprecision’ when the sample size was too small (<500 cases) or the confidential interval (CI) was too large. 3. Indirectness: We assigned ‘indirectness’ when the outcome was derived from study populations that differed from those of interest or key information of methods such as detection method or sample source are different. 4. lnconsistency: We assigned ‘lnconsistency’ by accounting heterogeneity measured by the I2 statistic (>50%), variability in point estimates, and extent of overlap in confidence intervals. In order to ensure the reliability of the results, if the study does not report heterogeneity, we will set the results as serious. 5. Publication bias: We assigned ‘detected’ when substantial asymmetry was observed in the funnel plot, or when the p value was <0.10 in Deek’s test or Egger’s test. If the inspection is not carried out, we also regard it as risky. In order to ensure the reliability of the results, if the study does not report biased results, we will set the results as serious. 6. Large magnitude of effect: We assigned ‘large magnitude’ when the relative risk or equivalent was >2. 7. Dose-response association: We assigned ‘dose-response’ when the effect size showed statistically significant lncrement per unit lncreases in adiposity indices. | | | | | | | | | | | |

Supplementary Table 5: PRISMA checklist

| **Section and Topic** | **Item #** | **Checklist item** | **Reported on page**  **number/section**  **name** |
| --- | --- | --- | --- |
| **TITLE** | | |  |
| Title | 1 | Identify the report as a systematic review. | Page 1 |
| **ABSTRACT** | | |  |
| Abstract | 2 | See the PRISMA 2020 for Abstracts checklist. | Page 1 |
| **INTRODUCTION** | | |  |
| Rationale | 3 | Describe the rationale for the review in the context of existing knowledge. | Introduction |
| Objectives | 4 | Provide an explicit statement of the objective(s) or question(s) the review addresses. | Introduction |
| **METHODS** | | |  |
| Eligibility criteria | 5 | Specify the inclusion and exclusion criteria for the review and how studies were grouped for the syntheses. | Methods |
| Information sources | 6 | Specify all databases, registers, websites, organisations, reference lists and other sources searched or consulted to identify studies. Specify the date when each source was last searched or consulted. | Methods |
| Search strategy | 7 | Present the full search strategies for all databases, registers and websites, including any filters and limits used. | Supplementary table 1 |
| Selection process | 8 | Specify the methods used to decide whether a study met the inclusion criteria of the review, including how many reviewers screened each record and each report retrieved, whether they worked independently, and if applicable, details of automation tools used in the process. | Methods |
| Data collection process | 9 | Specify the methods used to collect data from reports, including how many reviewers collected data from each report, whether they worked independently, any processes for obtaining or confirming data from study investigators, and if applicable, details of automation tools used in the process. | Methods |
| Data items | 10a | List and define all outcomes for which data were sought. Specify whether all results that were compatible with each outcome domain in each study were sought (e.g. for all measures, time points, analyses), and if not, the methods used to decide which results to collect. | Methods |
|  | 10b | List and define all other variables for which data were sought (e.g. participant and intervention characteristics, funding sources). Describe any assumptions made about any missing or unclear information. | Methods |
| Study risk of bias assessment | 11 | Specify the methods used to assess risk of bias in the included studies, including details of the tool(s) used, how many reviewers assessed each study and whether they worked independently, and if applicable, details of automation tools used in the process. | Methods |
| Effect measures | 12 | Specify for each outcome the effect measure(s) (e.g. risk ratio, mean difference) used in the synthesis or presentation of results. | Methods |
| Synthesis methods | 13a | Describe the processes used to decide which studies were eligible for each synthesis (e.g. tabulating the study intervention characteristics and comparing against the planned groups for each synthesis (item #5)). | Methods |
|  | 13b | Describe any methods required to prepare the data for presentation or synthesis, such as handling of missing summary statistics, or data conversions. | Methods |
|  | 13c | Describe any methods used to tabulate or visually display results of individual studies and syntheses. | Methods |
|  | 13d | Describe any methods used to synthesize results and provide a rationale for the choice(s). If meta-analysis was performed, describe the model(s), method(s) to identify the presence and extent of statistical heterogeneity, and software package(s) used. | Methods |
|  | 13e | Describe any methods used to explore possible causes of heterogeneity among study results (e.g. subgroup analysis, meta-regression). | Methods |
|  | 13f | Describe any sensitivity analyses conducted to assess robustness of the synthesized results. | NA |
| Reporting bias assessment | 14 | Describe any methods used to assess risk of bias due to missing results in a synthesis (arising from reporting biases). | Methods |
| Certainty assessment | 15 | Describe any methods used to assess certainty (or confidence) in the body of evidence for an outcome. | Methods |
| **RESULTS** | | |  |
| Study selection | 16a | Describe the results of the search and selection process, from the number of records identified in the search to the number of studies included in the review, ideally using a flow diagram. | Results; Figure 1 |
|  | 16b | Cite studies that might appear to meet the inclusion criteria, but which were excluded, and explain why they were excluded. | Results; Figure 1 |
| Study characteristics | 17 | Cite each included study and present its characteristics. | Results; Table 1-2 |
| Risk of bias in studies | 18 | Present assessments of risk of bias for each included study. | Results; Table 3-4; Supplementary tables 5-6 |
| Results of individual studies | 19 | For all outcomes, present, for each study: (a) summary statistics for each group (where appropriate) and (b) an effect estimate and its precision (e.g. confidence/credible interval), ideally using structured tables or plots. | Results; Table 3-4; Supplementary figure 1-10 |
| Results of syntheses | 20a | For each synthesis, briefly summarise the characteristics and risk of bias among contributing studies. | Results |
|  | 20b | Present results of all statistical syntheses conducted. If meta-analysis was done, present for each the summary estimate and its precision (e.g. confidence/credible interval) and measures of statistical heterogeneity. If comparing groups, describe the direction of the effect. | Results |
|  | 20c | Present results of all investigations of possible causes of heterogeneity among study results. | Results |
|  | 20d | Present results of all sensitivity analyses conducted to assess the robustness of the synthesized results. | NA |
| Reporting biases | 21 | Present assessments of risk of bias due to missing results (arising from reporting biases) for each synthesis assessed. | NA |
| Certainty of evidence | 22 | Present assessments of certainty (or confidence) in the body of evidence for each outcome assessed. | Results; Figure 3; Supplementary tables 7-8 |
| **DISCUSSION** | | |  |
| Discussion | 23a | Provide a general interpretation of the results in the context of other evidence. | Discussion |
|  | 23b | Discuss any limitations of the evidence included in the review. | Discussion |
|  | 23c | Discuss any limitations of the review processes used. | Discussion |
|  | 23d | Discuss implications of the results for practice, policy, and future research. | Discussion |
| **OTHER INFORMATION** | | |  |
| Registration and protocol | 24a | Provide registration information for the review, including register name and registration number, or state that the review was not registered. | Methods |
|  | 24b | Indicate where the review protocol can be accessed, or state that a protocol was not prepared. | Methods |
|  | 24c | Describe and explain any amendments to information provided at registration or in the protocol. | Methods |
| Support | 25 | Describe sources of financial or non-financial support for the review, and the role of the funders or sponsors in the review. | Discussion |
| Competing interests | 26 | Declare any competing interests of review authors. | Discussion |
| Availability of data, code and other materials | 27 | Report which of the following are publicly available and where they can be found: template data collection forms; data extracted from included studies; data used for all analyses; analytic code; any other materials used in the review. | Supplementary material |


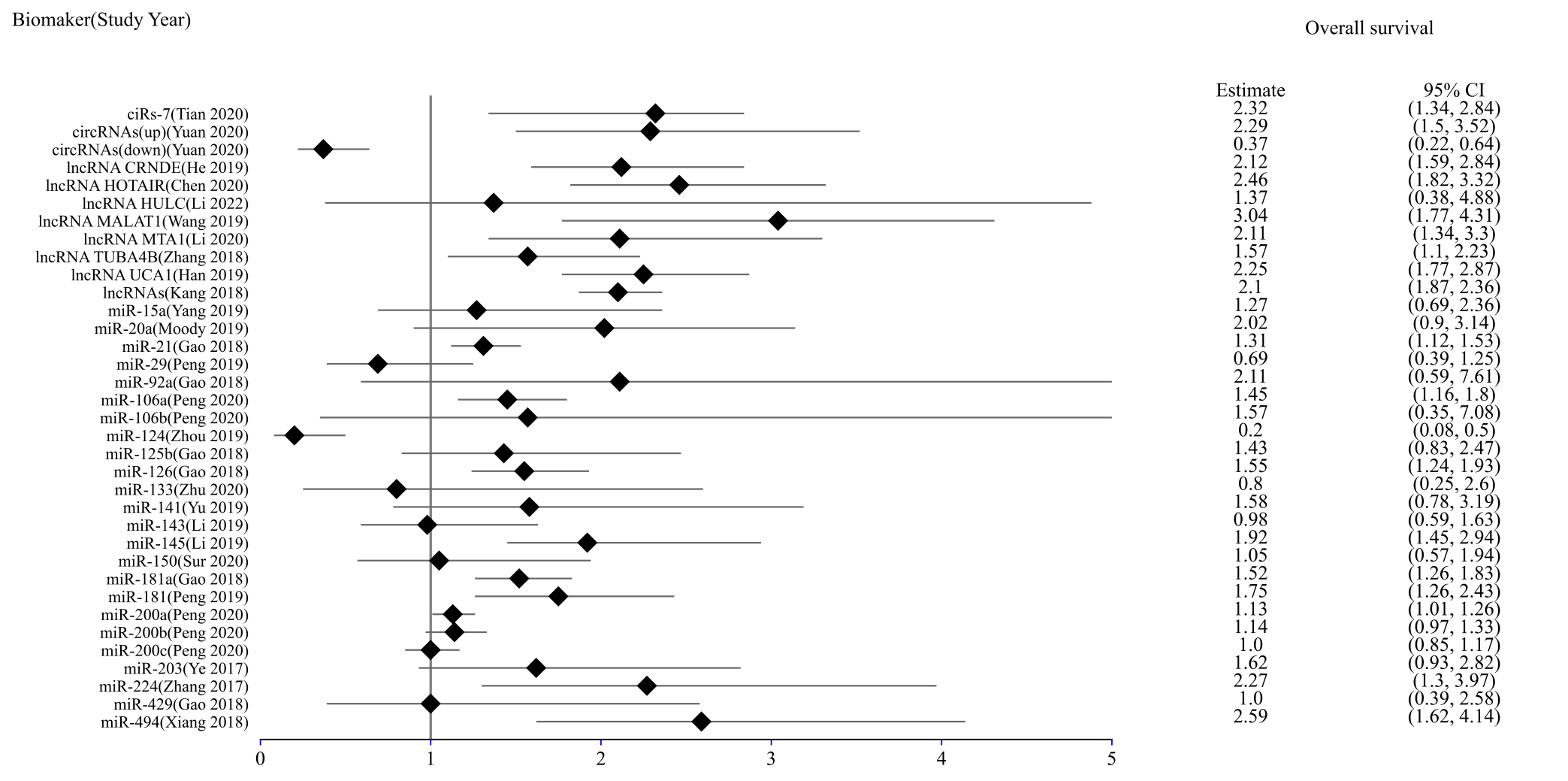
 Supplementary Figure 1: Main findings of the progonsis of ncRNA for CRC(Overall survival)


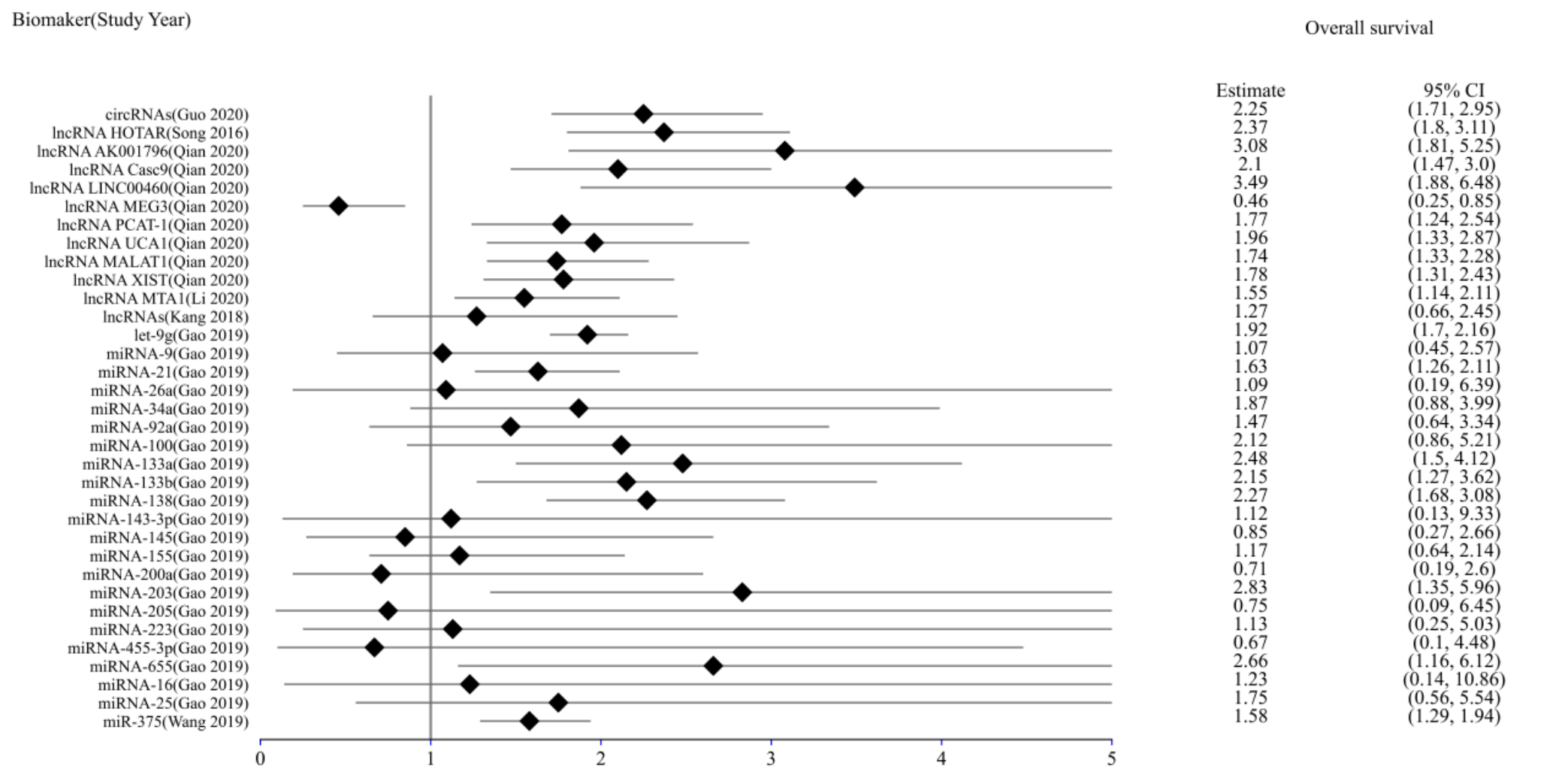


Supplementary Figure 2: Main findings of the progonsis of ncRNA for EC(Overall survival)


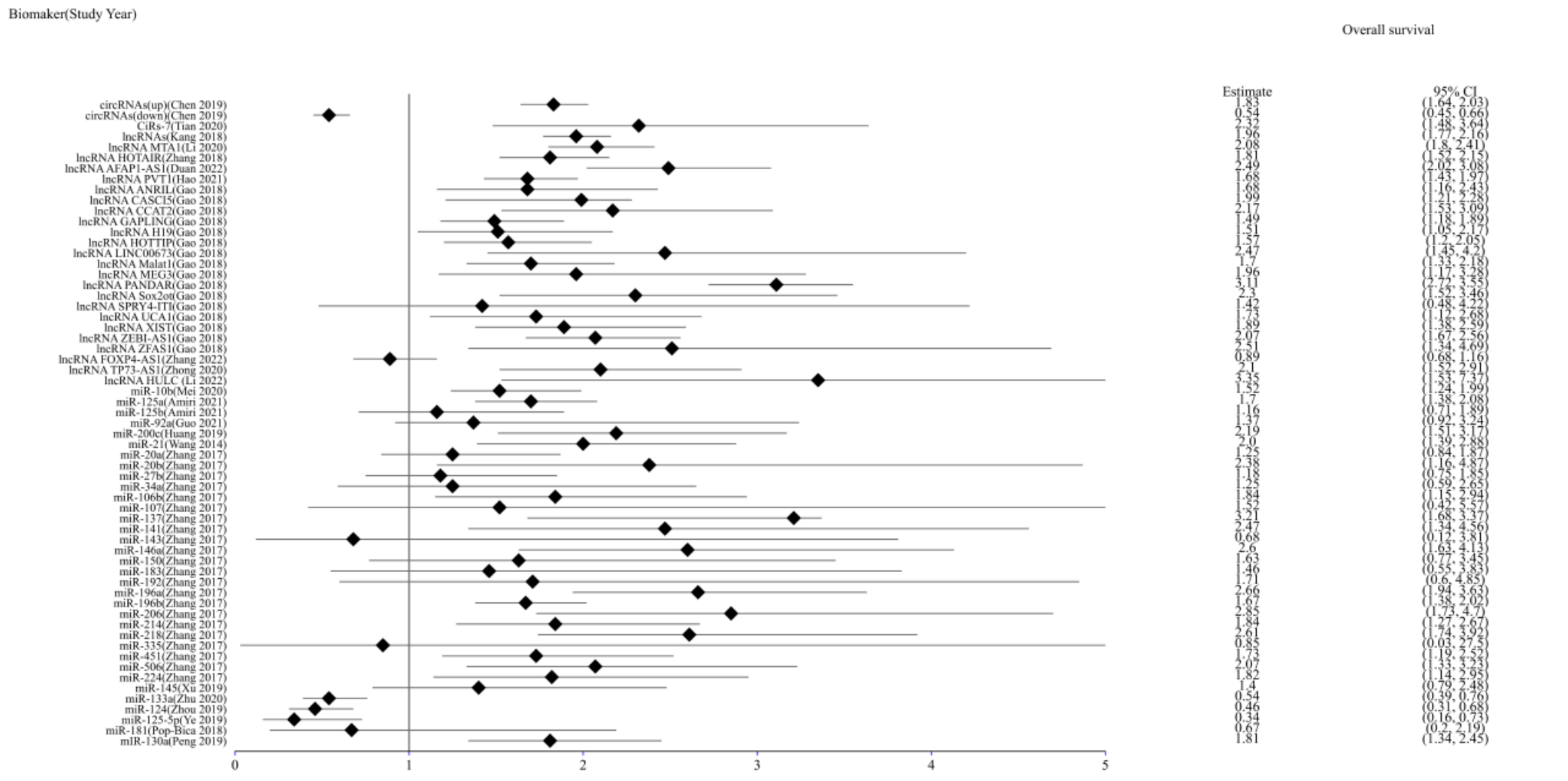


Supplementary Figure 3: Main findings of the progonsis of ncRNA for SC(Overall survival)


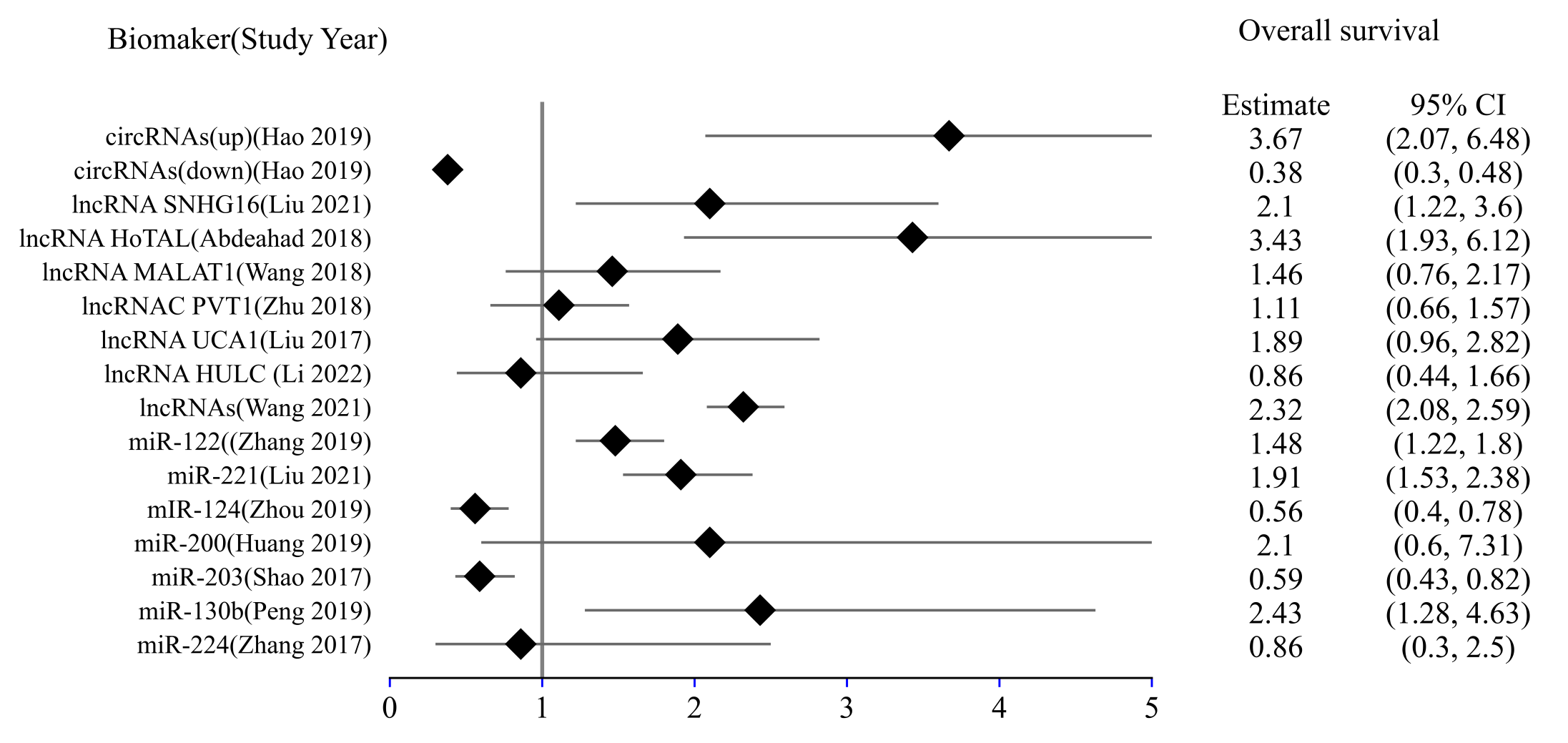


Supplementary Figure 4: Main findings of the progonsis of ncRNA for LC(Overall survival)


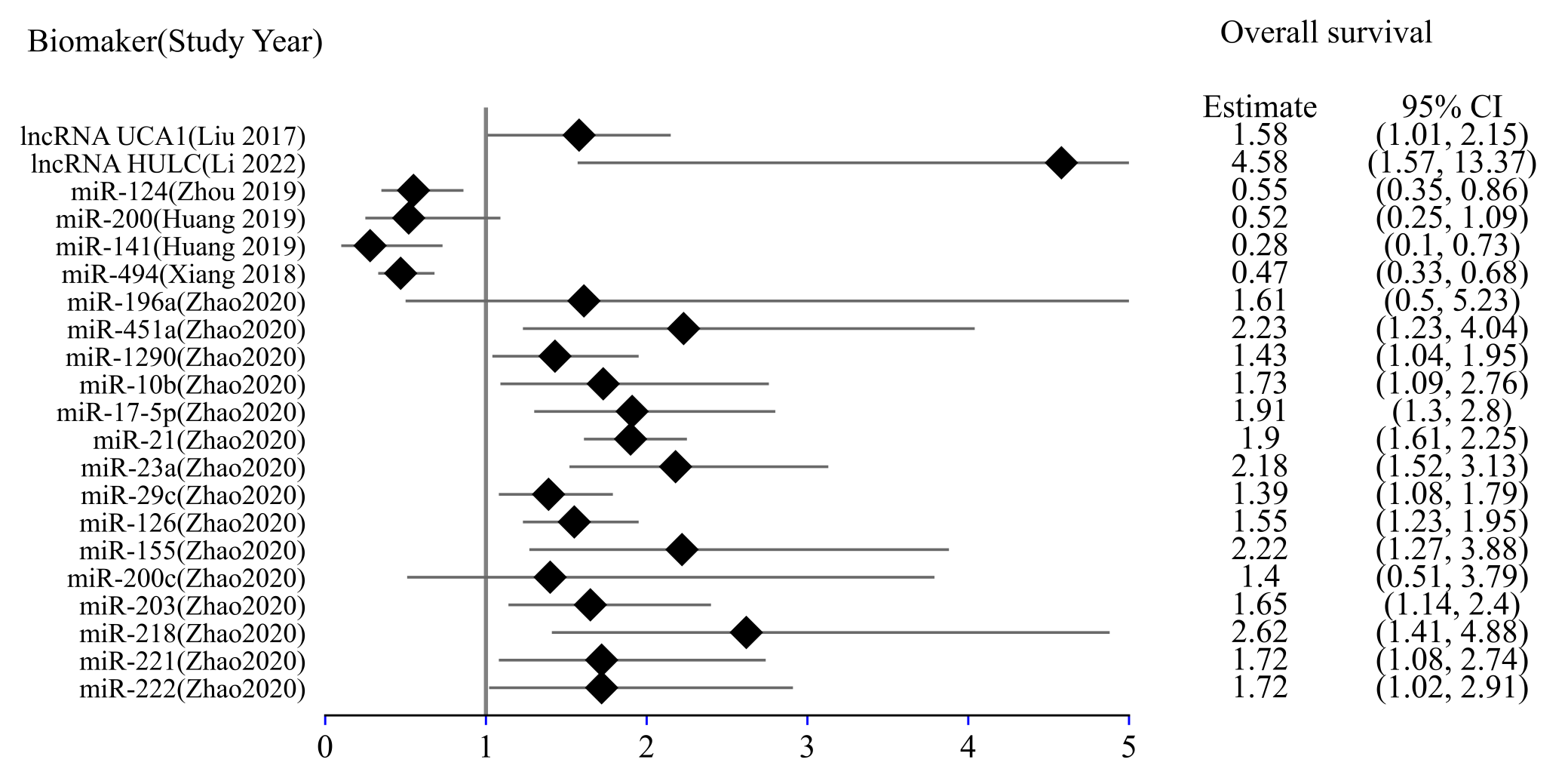


Supplementary Figure 5: Main findings of the progonsis of ncRNA for PC(Overall survival)
